# Uncovering structural variation in conifer gigagenomes: evolutionary insights and technical challenges

**DOI:** 10.1093/molbev/msag239

**Published:** 2026-09-18

**Authors:** Hui Liu, Wei Zhao, Jing-Fang Guo, Xue-Mei Yan, Yan-Jing Liu, Qing-Yin Zeng, Xiao-Ru Wang

**Affiliations:** Department of Ecology, Environment and Geoscience, Umeå Plant Science Centre, Umeå University, Umeå SE-90187, Sweden; Department of Ecology, Environment and Geoscience, Umeå Plant Science Centre, Umeå University, Umeå SE-90187, Sweden; Applied Research Center for Tropical Plant Conservation, Xishuangbanna Tropical Botanical Garden, Chinese Academy of Sciences, Mengla 666303, China; School of Horticulture and Food, Guangdong Eco-Engineering Polytechnic, Guangzhou 510520, China; State Key Laboratory of Vegetable Biobreeding, Institute of Vegetables and Flowers, Chinese Academy of Agricultural Sciences, Beijing 100081, China; State Key Laboratory of Tree Genetics and Breeding, Chinese Academy of Forestry, Beijing 100091, China; State Key Laboratory of Tree Genetics and Breeding, Chinese Academy of Forestry, Beijing 100091, China; Department of Ecology, Environment and Geoscience, Umeå Plant Science Centre, Umeå University, Umeå SE-90187, Sweden

**Keywords:** structural variants, functional effect, transposable elements, graph pangenome, bioinformatic artifacts, *Pinus*

## Abstract

Structural variants (SVs) are a major yet understudied source of genomic variation in conifers, whose large, repeat-rich genomes have hindered systematic SV discovery. Here, we combined whole-genome long-read and short-read sequencing to characterize the genomic landscape, functional impact, and evolutionary significance of SVs in a complex of three closely related pine species (*Pinus densata*, *Pinus tabuliformis*, and *Pinus yunnanensis*) with a hybridization history. From 21 long-read-sequenced individuals, we identified 5.7 million SVs, comprising 52% insertions, 43% deletions, and 5% inversions, duplications, and translocations. Approximately 97% of SVs were located in intergenic and intronic regions, and 60% overlapped transposable elements, whose activity shapes SV abundance and size variation. The proportion of loss-of-function (LoF) mutations was hundreds-fold higher among SVs than SNPs, with longer SVs more likely to cause LoF effects across all SV classes. Estimates of population diversity based on SVs and SNPs were largely concordant. In *P. densata*, the retention of parental SVs highlights the genomic signature of its admixed ancestry. We conducted graph pangenome-based SV genotyping in 29 short-read-sequenced individuals, yielding 44% recall and 70% precision, underlining the challenge of accurately recovering long-read-derived SVs in highly repetitive conifer genomes. Population-level selection scans on SNPs and genotyped SVs identified only 19% of candidate gene loci in common, indicating that the two marker types capture complementary components of environmental adaptation. Our findings demonstrate the importance of SVs as a dimension of genomic diversity and provide a foundation for integrating structural variation into evolutionary studies, conservation genomics, and tree breeding.

## Introduction

High-throughput sequencing has transformed evolutionary genomics by enabling genome-wide analyses based largely on single-nucleotide polymorphisms (SNPs), the predominant marker type of the past two decades. While SNPs have greatly advanced our understanding of genetic diversity and evolution, another major form of genetic variation, structural variants (SVs), remains poorly explored, especially in non-model plants. SVs, including larger-scale genomic alterations such as insertions (INS), deletions (DEL), inversions (INV), duplications (DUP), and translocations (TRA), constitute a substantial portion of genomic diversity as revealed in model organisms (Zhao et al. 2018; Alonge et al. 2020; Loegler et al. 2025; Logsdon et al. 2025). Because SVs can alter gene dosage, gene regulation, and linkage relationships, they often have larger functional effects than SNPs (Wellenreuther et al. 2019; Alonge et al. 2020; Todesco et al. 2020; He et al. 2025). Genome-wide SVs may therefore uncover population structure, trait genetics, and signals of selection and local adaptation that remain undetected in SNP-based analyses (Dorant et al. 2020; Merot et al. 2020; Hämälä et al. 2021; Liu et al. 2024b; Gompert et al. 2025; Liang et al. 2025).

Current knowledge of structural variation in plant genomes is derived primarily from crop species (Shang et al. 2022; Zhou et al. 2022; Zhang et al. 2024a; Liu et al. 2024b; Zhang et al. 2024b; Guo et al. 2025), whereas studies of trees, particularly conifers, remain scarce. Available reports on broadleaf trees show marked differences in SV abundance among species (Hämälä et al. 2021; Chen et al. 2023; Shi et al. 2024; Liang et al. 2025; Li et al. 2025a), likely attributable to genome size, transposable element (TE) activity, and methodological differences. Conifer genomes, which often exceed 20 Gb, are dominated by repetitive sequences, particularly long terminal repeat (LTR) retrotransposons (Nystedt et al. 2013; Stevens et al. 2016; Jang et al. 2024; Neale et al. 2024). These features have long hindered accurate SV detection using short-read sequencing, restricting population studies almost exclusively to SNPs. As a result, the extent, genomic distribution, and evolutionary significance of SVs in conifers remain largely unknown. Recent advances in long-read sequencing technologies and SV-calling algorithms (De Coster et al. 2019; Mahmoud et al. 2019; Liu et al. 2024a) now make it feasible to identify and characterize SVs even in large, repeat-rich genomes.

In this study, we use long-read sequencing and graph-based SV genotyping to provide the first population-scale characterization of structural variation in conifers. We focus on three closely related pine species, *Pinus densata, Pinus tabuliformis*, and *Pinus yunnanensis*, whose evolutionary history has been investigated using SNPs from exome capture sequencing (Zhao et al. 2020; Guo et al. 2023) and cytoplasmic DNA markers (Song et al. 2003; Wang et al. 2011). *Pinus densata* is distributed across the southeastern Tibetan Plateau at elevations of 2,700 to 4,200 meters above sea level (Mao and Wang 2011). Converging evidence from molecular and morphological data suggests that *P. densata* originated through hybridization between *P. tabuliformis* and *P. yunnanensis* in the late Miocene (Wang and Szmidt 1994; Mao et al. 2009; Wang et al. 2011; Gao et al. 2012). The distribution of these three species forms a geographic succession from north (*P. tabuliformis*) to southwest China (*P. yunnanensis*), with *P. densata* occupying an intermediate range (Mao and Wang 2011). Divergence between *P. tabuliformis* and *P. yunnanensis* is estimated at 15 to 20 million years (Gao et al. 2012; Jin et al. 2021; Zhao et al. 2024). An ancient hybrid zone between the two species at the northeastern margin of *P. densata*'s range is thought to have given rise to *P. densata*, which later expanded westward (Wang et al. 2011; Gao et al. 2012; Zhao et al. 2020). This well-characterized species complex, supported by a high-quality reference genome for *P. tabuliformis* (Niu et al. 2022), provides a valuable system to investigate structural diversity, divergence, and inheritance among closely related pine species.

Specifically, we (i) characterize the genomic distribution, diversity, and TE association of SVs in pine genomes; (ii) compare the functional effects of SVs and SNPs, and their contribution to population differentiation and species divergence; and (iii) evaluate SV-calling strategies and graph-based SV detection for analyzing large complex genomes. Our study provides an overview of structural variation in pine, uncovering previously uncharted dimensions of genomic diversity that complement SNP-based information and establishing a foundation and roadmap for future studies of adaptation, conservation, and tree breeding.

## Results

### SV discovery, diversity, and distribution in pine genomes

To capture SVs in pine genomes, we generated Oxford Nanopore Technologies (ONT) long-read sequences for 21 individuals from *P. densata* (Pd), *P. tabuliformis* (Pt), and *P. yunnanensis* (Py) (Fig. 1a and Table S1), yielding 211 to 323 Gb reads per individual with an N50 of 21 to 40 kb (Table S1). Reads were mapped to the chromosome-level *P. tabuliformis* reference genome (Niu et al. 2022) for SV detection, achieving mapping rates >99% at MAPQ (mapping quality) >0 and 77% to 83% at MAPQ ≥20 in all samples (Table S2). Median sequencing depths were 8.7×, 11.4×, and 9.4× for Pt, Pd, and Py, respectively, with comparable depth distribution among chromosomes (Fig. S1).

**Figure 1:**
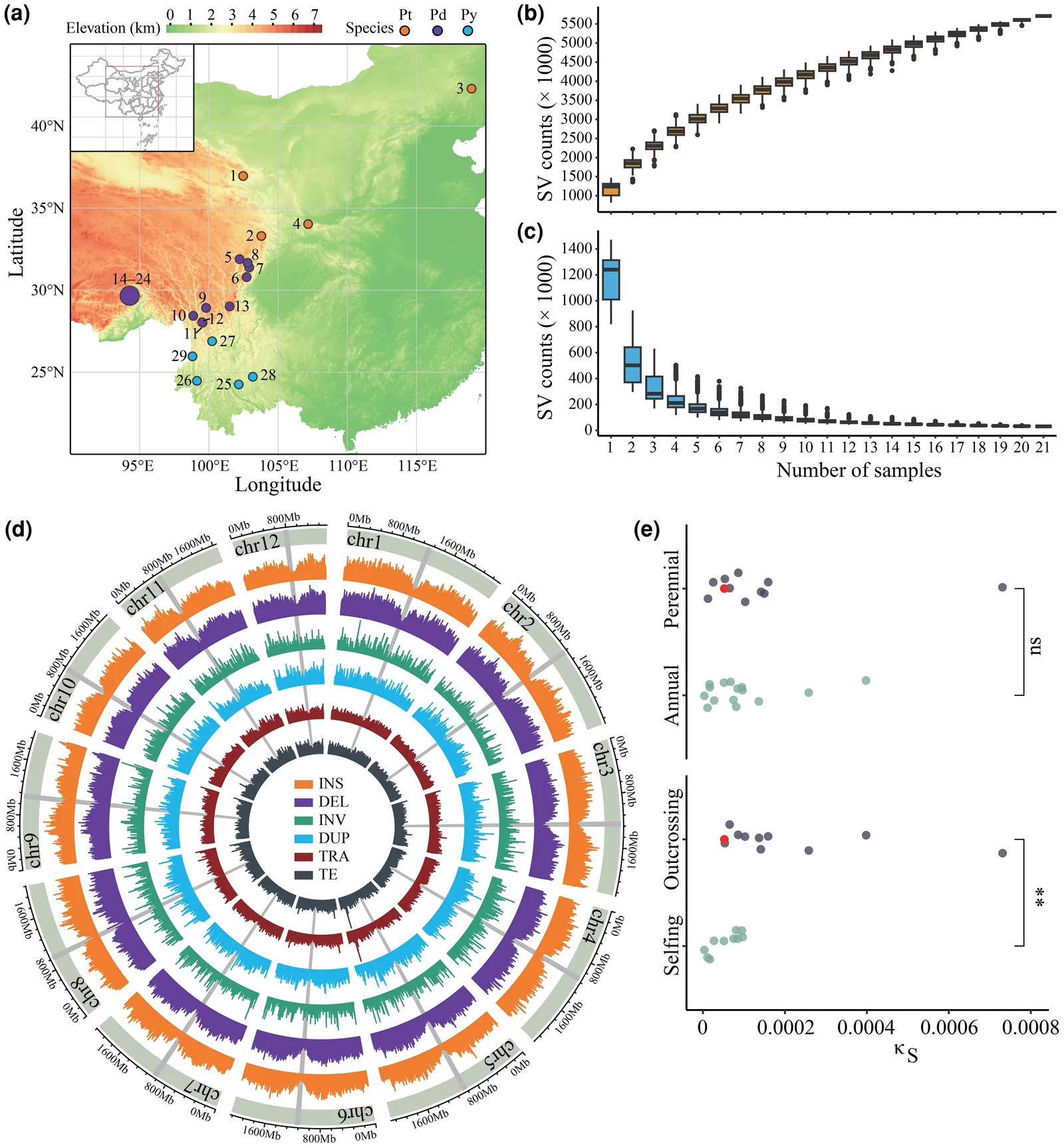
SV discovery and genomic distribution in pine. a) Locations of the samples. Pt, *Pinus tabuliformis*; Pd, *P. densata*; Py, *P. yunnanensis*. b and c) Accumulation of pan-SVs and core-SVs across increasing numbers of samples. Order of samples being added into the pan-SVs and core-SVs with 1,000 times bootstraps. d) Distribution of SVs and TEs across the genome. The tracks from the outer to the inner circle represent the 12 chromosomes (chr1 to chr12) of *P. tabuliformis* reference genome, the density of insertions (INS), deletions (DEL), inversions (INV), duplications (DUP), translocations (TRA), and TEs along 5 Mb nonoverlapping windows. The shadow bar represents the putative centromeric region. e) Structural diversity (κ_S_) in plants, computed as Watterson’s estimator. Original data were retrieved from 25 published studies (see Table S8). The red dot represents the pine of this study. Pairwise Wilcoxon tests were used to assess differences between groups (ns, not significant; **P < 0.01).

Because no prior knowledge exists on optimal SV detection strategies for conifer genomes, we first tested four callers on the 21 ONT-sequenced samples using identical SV merging parameters (see Materials and methods). The four callers recovered similar numbers of nonredundant SVs (4.5 to 5.0 million; Table S3). We selected Sniffles (Smolka et al. 2024) for its joint-calling framework suited for population genetic analyses. Because equivalent SVs across samples may be represented with slightly different breakpoint coordinates, lengths, or insertion sequences, accurately matching and merging variants became challenging. We therefore optimized the SV-merging criteria by evaluating 24 sets of parameter combinations in Truvari (English et al. 2022) across different SV types, considering breakpoint distance (refdist), size similarity (pctsize), and sequence similarity (pctseq) among variants (Table S4). Both SV abundance and allele frequency spectra were strongly affected by these parameters (Fig. S2; Tables S4 and S5). Increasing pctsize and pctseq thresholds progressively raised the number of nonredundant SVs from 4.5 million under the most relaxed setting (--refdist 500 --pctsize 0 --pctseq 0) to 5.8 million under the strictest (--refdist 500 --pctsize 1.0 --pctseq 1.0). This increase was largely contributed by INS and DEL, particularly low-frequency (≤ 5%) variants (INS: 34.9% to 46.7%; DEL: 31.6% to 36.8%) (Tables S4 and S5), suggesting that more stringent matching criteria reduced the collapsing of distinct alleles during SV merging. INV, DUP, and TRA were insensitive to these parameters, and reducing refdist from 500 to 50 bp had only a minor effect on SV calls (Tables S4 and S5**)**. Considering the combined effects of all tested parameters, we selected --refdist 500 --pctsize 0.9 --pctseq 0.9 for all downstream analyses. This parameter combination yielded a final set of 5,709,223 nonredundant SVs, with 820,023 to 1,470,844 SVs per individual (Table 1 and Table S6**)**.

**Table 1:** Summary of SV discovery using Oxford Nanopore Technologies (ONT) long-reads and a joint-call procedure in *Pinus densata* (Pd), *P. tabuliformis* (Pt), and *P. yunnanensis* (Py).

Species (N)	All SVs	INS	DEL	INV	DUP	TRA
Pd E (4)	2,935,511	1,404,976 (47.9%)	1,425,799 (48.6%)	3,875 (0.1%)	11,220 (0.4%)	89,641 (3.1%)
Pd C (5)	2,844,074	1,367,641 (48.1%)	1,342,088 (47.2%)	3,686 (0.1%)	12,114 (0.4%)	118,545 (4.2%)
Pd W (3)	2,098,685	976,231 (46.5%)	1,028,463 (49.0%)	2,617 (0.1%)	7,630 (0.4%)	83,744 (4.0%)
All Pd (12)	4,641,753	2,333,931 (50.3%)	2,057,023 (44.3%)	6,367 (0.1%)	22,378 (0.5%)	222,054 (4.8%)
Pt (4)	2,129,668	998,653 (46.9%)	1,079,664 (50.7%)	2,740 (0.1%)	7,291 (0.3%)	41,320 (1.9%)
Py (5)	2,563,597	1,233,489 (48.1%)	1,229,549 (48.0%)	3,232 (0.1%)	9,600 (0.4%)	87,727 (3.4%)
Total (21)	5,709,223	2,959,992 (51.8%)	2,435,205 (42.7%)	7,985 (0.1%)	29,337 (0.5%)	276,704 (4.8%)

Pd samples were divided into eastern (Pd E), central (Pd C), and western (Pd W) groups. INS, insertions; DEL, deletions; INV, inversions; DUP, duplications; TRA, translocations.

To verify the accuracy of this callset, we manually inspected long-read alignments of 20 randomly selected INS and DEL across five samples (total 100 cases) using Integrative Genomics Viewer (IGV) (Thorvaldsdottir et al. 2013), confirming an overall SV-calling accuracy of 88.0% and a validation rate of 71.9% for heterozygous (0/1) genotypes (Fig. S3; Table S7).

We examined the accumulation of pan-SVs in the 21 samples by treating them as a species complex given their close evolutionary relationships (Wang et al. 2011; Zhao et al. 2020, 2024; Guo et al. 2023). As sample size increased, the cumulative number of pan-SVs continued to climb, although the rate of discovery gradually slowed, indicating that SV diversity remained unsaturated even at 21 samples (Fig. 1b). In contrast, core-SVs (ie those shared by all samples, as either heterozygous [0/1] or homozygous alternative [1/1] genotypes) plateaued around 15 samples, at an average of 48,814 SVs with only a slight decline thereafter (Fig. 1c).

Within each species, 4.6, 2.6, and 2.1 million SVs were identified in Pd, Py, and Pt, respectively (Table 1). The larger number of SVs detected in Pd was primarily attributable to its larger sample size. When sample size was standardized to four individuals, 2.8, 2.3, and 2.1 million SVs were retrieved in Pd, Py, and Pt, respectively (Fig. S4). The remaining difference likely reflects the hybrid origin of Pd and between-species divergence associated with cross-species reference mapping (see later section).

SVs were divided into five classes, INS, DEL, DUP, INV, and TRA, which differed markedly in abundance and size distributions. In this study, INS and DEL were defined relative to the reference genome. INS (51.8%) and DEL (42.7%) were the most abundant, followed by TRA (4.8%), DUP (0.5%), and INV (0.1%) (Table 1). The median lengths of INS (174 bp) and DEL (263 bp) were significantly shorter than those of INV (15,020 bp) and DUP (17,604 bp) (Fig. S5). All classes were spread out across all 12 chromosomes, with reduced density around centromeric regions (Fig. 1d and Fig. S6). The average genome-wide SV density was 224 SVs per Mb. We calculated Watterson’s diversity estimator for SVs (κ_S_) (Loegler et al. 2026), which represents the number of SVs per base pair in a population. For pines, this was 5.22 × 10^−5^. To compare structural diversity among plant species, we retrieved SV datasets from 25 published studies that included at least 15 individuals each (Table S8) and computed the κ_S_ for each species (or species complex). We found that pine exhibits relatively low structural diversity among outcrossing perennial plants (Fig. 1e).

### Association with TEs

Given the high repetitiveness of the pine genome, we expected TEs to strongly impact SV dynamics. A sliding window analysis showed a positive correlation between TE content and SV frequency genome-wide (Spearman's ρ = 0.42, *P* < 0.001; Fig. 2a). Repeat annotation of SV breakpoint windows (±50 bp) using a 60% minimum-overlap criterion revealed that 62.8% of SVs overlapped with repeat elements, most of which were TEs (59.7%; Fig. 2b). DUP had the highest overlap with repeat elements (73.0%), while other classes ranged from 61.0% (INS) to 66.0% (INV). The LTR retrotransposon superfamilies *Gypsy* and *Copia* comprised 36.7% of all SV breakpoints, and this proportion was even higher (43.7% to 50.6%) for TRA, INV, and DUP (Fig. 2b). In pine, LTRs account for ∼60% of the genome (Niu et al. 2022), making the frequent overlap of SV breakpoints with LTRs unsurprising. These results provide a quantitative assessment of SV-TE associations and illustrate the contribution of LTR retrotransposons to shaping the genomic landscape of SVs.

**Figure 2:**
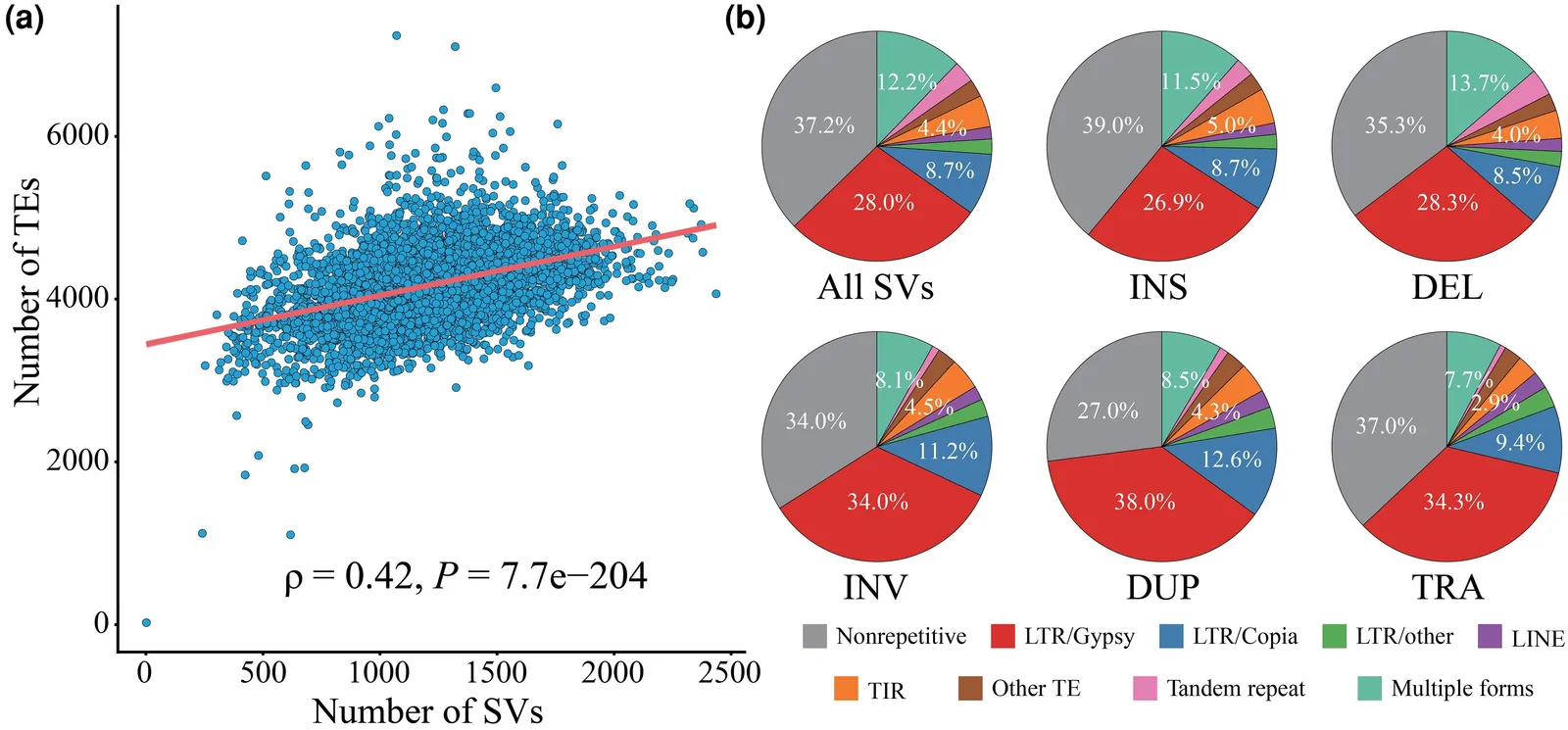
Association of SVs with repeat elements. a) Correlations between SVs and TEs along 5 Mb nonoverlapping windows. b) Proportion of different classes of SVs overlapping with repeat elements. LTR, long terminal repeat; LINE, long interspersed nuclear element; TIR, terminal inverted repeat; TE, transposable element. Multiple forms, repeats with mixed types.

### Genomic context and functional effect of SVs

To assess the functional properties of the 5.7 million SVs, we annotated each variant by the genomic locations of its breakpoints, and classified them into coding sequence (CDS), untranslated region (UTR), intronic, regulatory (within 2 kb upstream or downstream of genes), and intergenic (excluding the regulatory regions) categories. The majority of SVs (97.3%) were located in intergenic and intronic regions, while the remaining 2.7% overlapped genic and regulatory regions (Fig. 3a). Of these, 47,789 SVs (0.8%) were classified as loss-of-function (LoF) variants, representing a proportion approximately 400-fold higher than that observed for SNPs (0.002%; Fig. 3b). TRA exhibited the highest LoF proportion (10.1%), whereas DUP showed the lowest. LoF SVs were also significantly longer than non-LoF SVs within each SV class (Fig. 3c; Wilcoxon tests, *P* < 0.0001).

**Figure 3:**
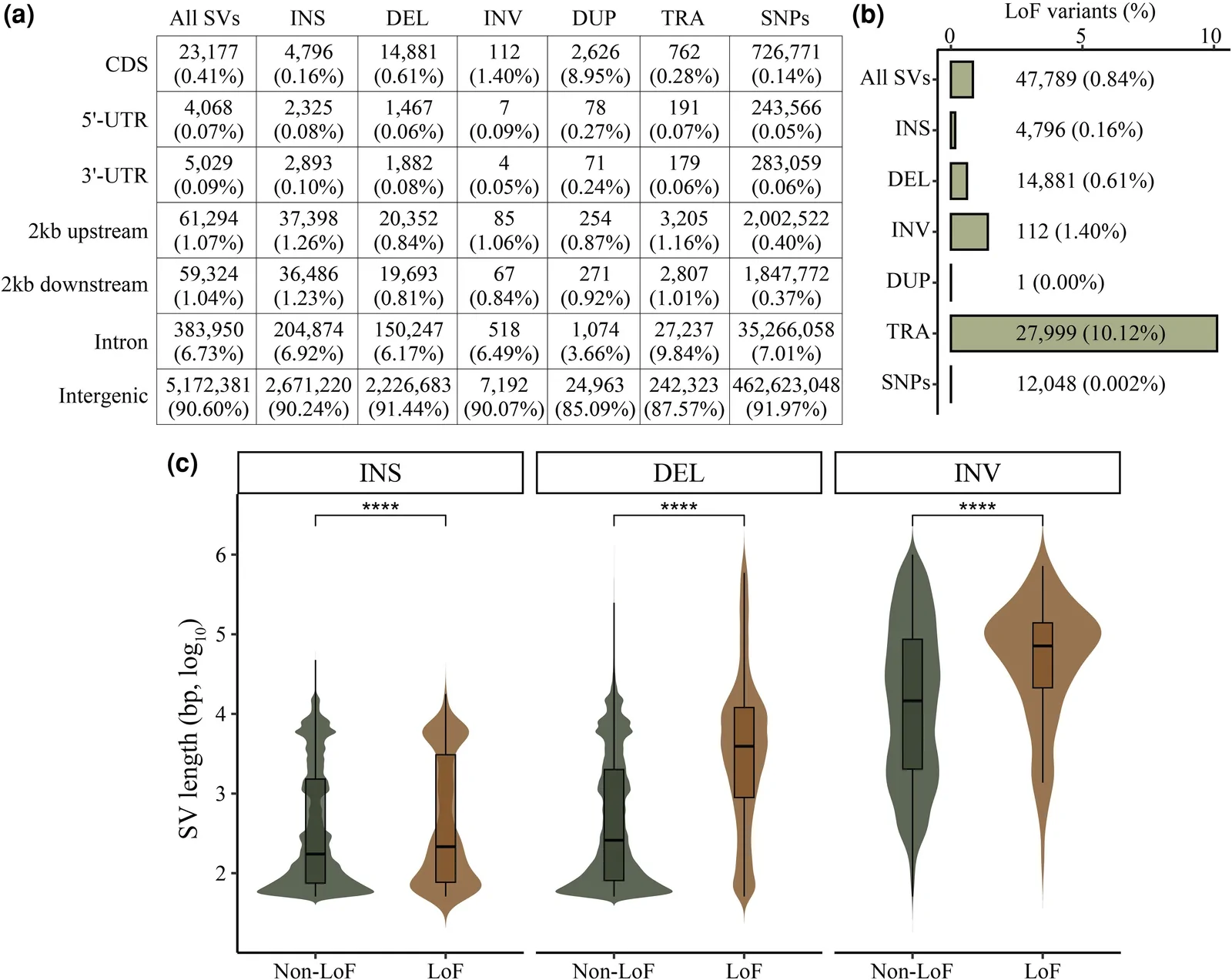
Genomic annotation of SVs and SNPs from 21 pine samples. a) Distribution of SVs and SNPs across genomic regions. Numbers indicate the counts and percentages of variants in each category. SNPs were filtered with a missing rate ≤20% across samples. b) Proportion of loss-of-function (LoF) variants for SVs and SNPs. c) Length distribution of LoF and non-LoF SVs across SV types. Pairwise Wilcoxon tests were used to assess differences between groups (****P < 0.0001).

### Genetic diversity measured by SVs and SNPs

To compare patterns of population genetic structure and diversity inferred from SVs and SNPs, we generated a SNP dataset using Illumina short reads (258 to 748 Gb per sample) for the same 21 ONT-sequenced samples (Table S1). Filtering for missing genotype rate <20% and a minor allele count ≥ 3 (M20.mac3) retained 1,688,063 SVs and 183,104,659 SNPs, and further linkage disequilibrium (LD) pruning reduced the SV and SNP datasets to 150,010 and 8,595,121 variants, respectively. These pruned datasets were used for principal component analysis (PCA) and ADMIXTURE to explore sample relationships.

Both datasets showed highly comparable differentiation patterns (Fig. 4a to d), each resolving five groups within the species complex: three geographical groups of *P. densata* (eastern, central, and western; Pd E, Pd C, and Pd W) and the two parental species, Pt and Py. The only notable difference was that SNP-based PCA failed to distinguish two Pd E individuals from Pt (Fig. 4b), whereas SV-based PCA clearly distinguished them. Because the two datasets differ in variant number by orders of magnitude, we subsampled the SNP dataset to match the SV site frequency distribution to exclude this as a confounding factor; the resulting PCA was virtually identical to that from the full SNP dataset (Fig. S7).

**Figure 4:**
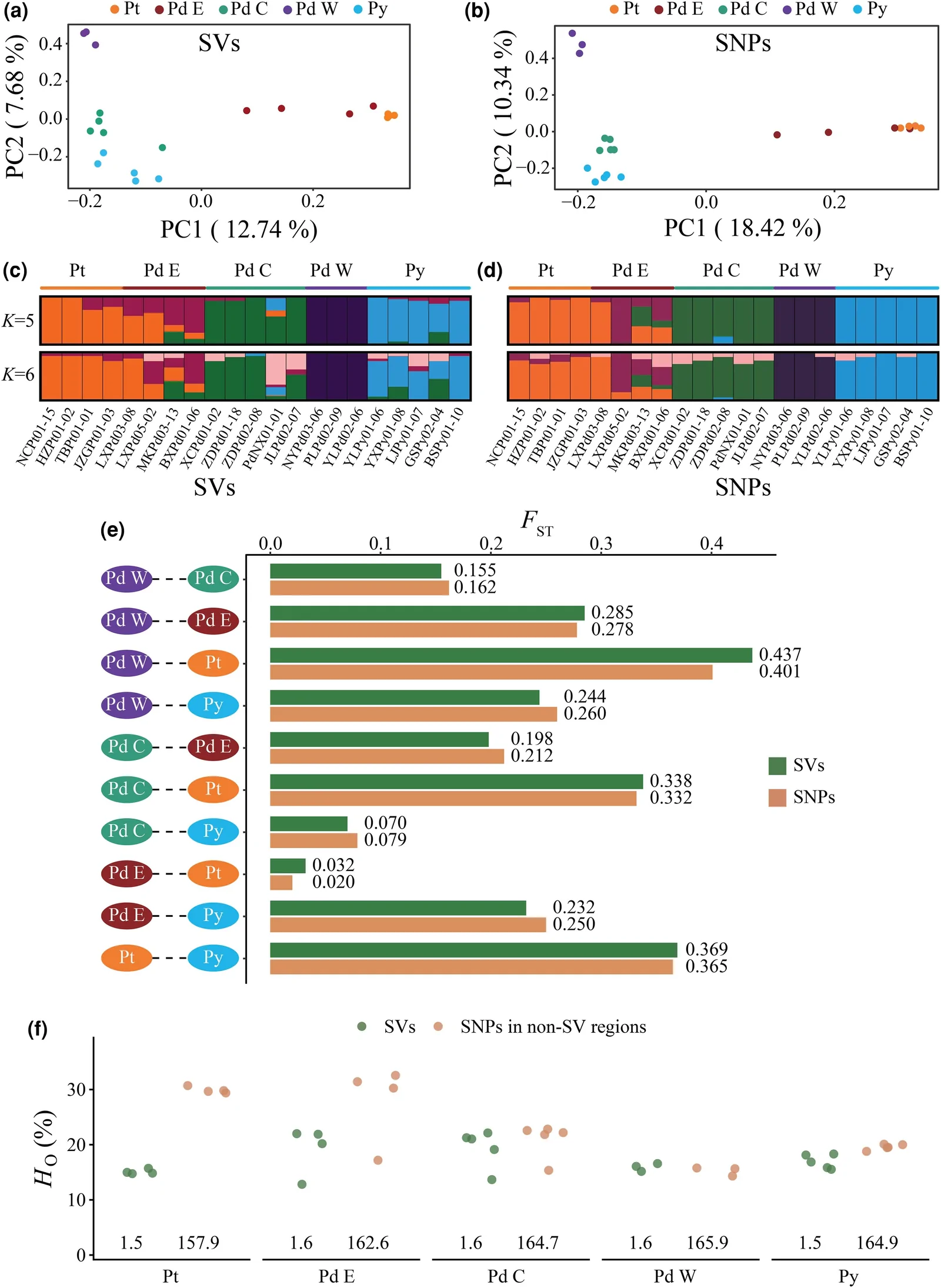
Genetic diversity measured by SVs and SNPs. a and b) PCA plot showing the first two principal components (PC) for SVs and SNPs. The proportion of variance explained by each PC is shown in parentheses. c and d) Patterns of ADMIXTURE based on SVs and SNPs. e) Pairwise differentiation (*F*_ST_) between groups based on SVs and SNPs. f) Individual observed heterozygosity (*H*_O_) by SVs and SNPs in each sample. Numbers at the bottom indicate the mean number of SVs or SNPs in millions for each dataset.

Similarly, the SV-based ADMIXTURE corroborated the SNP patterns (Fig. 4c and d; Fig. S8), and pairwise genetic differentiation (*F*_ST_) among the five sample groups were comparable between the two datasets as well (Fig. 4e). On average, both SVs and SNPs yielded an *F*_ST_ 0.24 among the five groups, with highest differentiation between Pd W and Pt (*F*_ST_ = 0.44 for SVs, 0.40 for SNPs) and lowest between Pd E and Pt (0.03 for SVs and 0.02 for SNPs).

We also compared individual observed heterozygosity (*H*_o_) estimated from SVs and SNPs. Individual *H*_o_ ranged from 0.13 to 0.22 for SVs and 0.14 to 0.33 for SNPs (Fig. 4f). Estimates from the two marker types were generally concordant, except for a few Pt and Pd E individuals, which showed lower *H*_o_ based on SVs than SNPs. Further examination revealed a positive correlation between SV-based *H*_o_ and sequencing depth, whereas no such relationship was observed for SNPs (Fig. S9). This pattern likely reflects the reduced sensitivity for detecting heterozygous SV genotypes at low sequencing depth relative to SNP calling.

### Hybridization legacy preserved in SVs

As presented earlier, Pd harbored more SVs than Pt and Py even after eliminating sample size differences. At species level, Pd shared 1,066,617 and 526,921 pairwise-specific SVs with Py and Pt, respectively, much larger than the 40,919 SVs shared between Pt and Py, and a total of 995,669 SVs were shared among all three species (Fig. 5a). To test whether this extensive overlap among the three species could have arisen by chance, we performed permutation tests (n = 10,000), which showed that the observed number of all-species-shared SVs was significantly greater than expected by chance (*P* < 1 × 10^−4^), consistent with the inheritance and retention of parental SVs following the hybrid origin of *P. densata*. These all-species-shared SVs were distributed across all 12 chromosomes (Fig. S10) and closely mirrored the genomic landscape of the full set of SVs (Fig. S6; Spearman's ρ = 0.78, *P* < 0.001).

**Figure 5:**
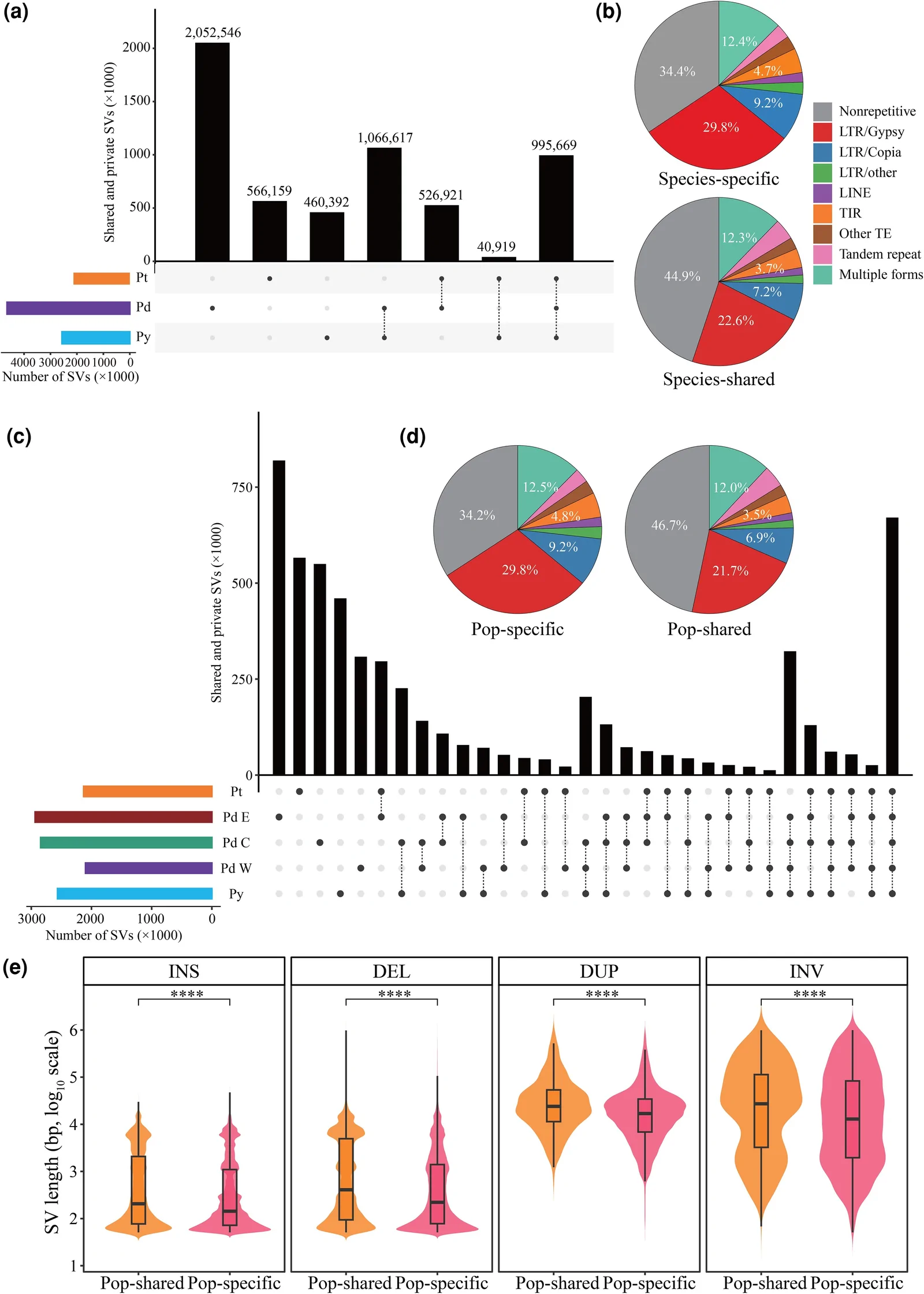
Properties of species-specific and species-shared SVs in the species complex. a) Number of species-specific and shared SVs between *P. densata* and parental species *P. tabuliformis* and *P. yunnanensis*. b) Composition of TEs and other sequence forms in species-specific and all-species-shared SVs. c) Number of population-specific and shared SVs across five sample groups. d) Composition of TEs and other sequence forms in population-specific and shared SVs. e) Length distribution of population-specific and shared SVs across SV types. Pairwise Wilcoxon tests were used to assess differences between groups (****P < 0.0001). Population-shared SVs refer to variants present in all five sample groups.

The ∼1 million all-species-shared SVs overlapped 14,554 genes (ie genes overlapped by at least one shared SV in either CDS, introns, or UTRs) of all essential housekeeping biological categories (Supplementary Data S1). Gene Ontology (GO) analysis revealed a significant enrichment of core genes involved in fundamental cellular processes, including tRNA and rRNA metabolic processes, tRNA aminoacylation, RNA methylation, and regulatory pathways such as protein import and targeting and transcription regulator complexes (Figs. S11 to S13; Supplementary Data S2).

In contrast to the biological properties retained in species-shared SVs, species- or population-specific SVs showed a distinct profile (Fig. 5b to e): they had stronger associations with repetitive elements (eg 65.6% in species-specific vs. 55.1% in species-shared SVs), particularly LTR (*Gypsy* and *Copia*) elements, and shorter length in each SV class (Fig. 5e and Fig. S14). Given that LTR insertion activity in *P. tabuliformis* peaked at ∼6 million years ago (Niu et al. 2022; Bao et al. 2026), we interpret these features as suggestive of relatively recent origin for population-specific SVs, although this interpretation assumes that TE dynamics are comparable across the examined species. We also note that the small per-population sample sizes may lead to incomplete detection of low-frequency SVs and thus misclassification of population-specific and population-shared variants. Larger samples and explicit frequency-based detection modeling would help clarify these interesting observations.

### Graph-based SV genotyping at population scale

The high cost of long-read sequencing limits the number of individuals that can be sequenced and analyzed, particularly for conifers with large genomes. A common strategy is to first construct a high-confidence SV catalog using long reads and then genotype these variants in a larger population of short-read-sequenced individuals for evolutionary inference. In this study, we constructed a graph pangenome by integrating the *P. tabuliformis* reference genome with 5,401,395 SVs, including 2,958,205 sequence-resolved INS, 2,435,205 DEL, and 7,985 INV, using vg (Garrison et al. 2018). DUP, TRA, and sequence-unresolved INS were excluded because they are not supported for genotyping by vg. The resulting graph is 31.0 Gb, an increase of 5.6 Gb relative to the 25.4 Gb reference genome.

We then evaluated the ability of graph-based SV-calling using short reads to recover the known variants from the same set of 21 samples used to construct the SV catalogue. This design provides a measure of SV recall and precision against a long-read–derived benchmark. The graph-based analysis achieved a high mapping rate (∼97%, Table S9) to the pangenome and identified SVs with a precision of 70% (Table S10). However, only an average of 44% of long-read-defined SVs were recovered per sample, yielding an overall F1 score (the harmonic mean of precision and recall) of 0.54 (Table S10), indicating that many SVs identified by long reads remain challenging to genotype accurately using short reads.

Next, we applied this approach to an independent population dataset. We generated short-read sequences for 11 individuals from population Pd W (266 to 468 Gb per individual; Table S1) and performed graph-based SV genotyping (Table S9). Focusing on a single population eliminates potential confounding of population structure on allele frequency estimation and selection inference (Fig. S15). Genotypes were successfully obtained for 1,638,632 SVs using the vg pipeline. For comparison, we also generated a SNP set for the same 11 individuals using the short reads. After filtering for a missing rate <20%, a minor allele count ≥1, and heterozygosity ≤50% (M20.mac1.het50), a total of 397,805 SVs and 122,655,565 SNPs were retained for minor allele frequency (MAF), annotation, and selection analyses. Similar to the complete SVs dataset, the graph-genotyped SVs in Pd W were strongly enriched in intergenic and intronic regions (Fig. 6a), and the predicted LoF mutations were more frequent among SVs than SNPs (0.25% vs. 0.003%; Fig. 6b). We examined the potential fitness effects of SVs by looking into their MAF distributions. Because graph-genotyped INV were rare, only INS and DEL were included in this analysis. INS and DEL annotated to different genomic features showed highly similar MAF distributions, with low-frequency variants (MAF ≤10%) accounting for 41.7% to 52.6% of SVs across genomic features (Fig. 6c). By comparison, SNPs showed a stronger excess (55.8% to 57.2%) of rare alleles, particularly for nonsynonymous SNPs (62.3%) (Fig. 6d). Relative to SNPs, INS and DEL exhibited a weaker skew toward low-frequency alleles, with INS exhibiting a slightly stronger skew than DEL.

**Figure 6:**
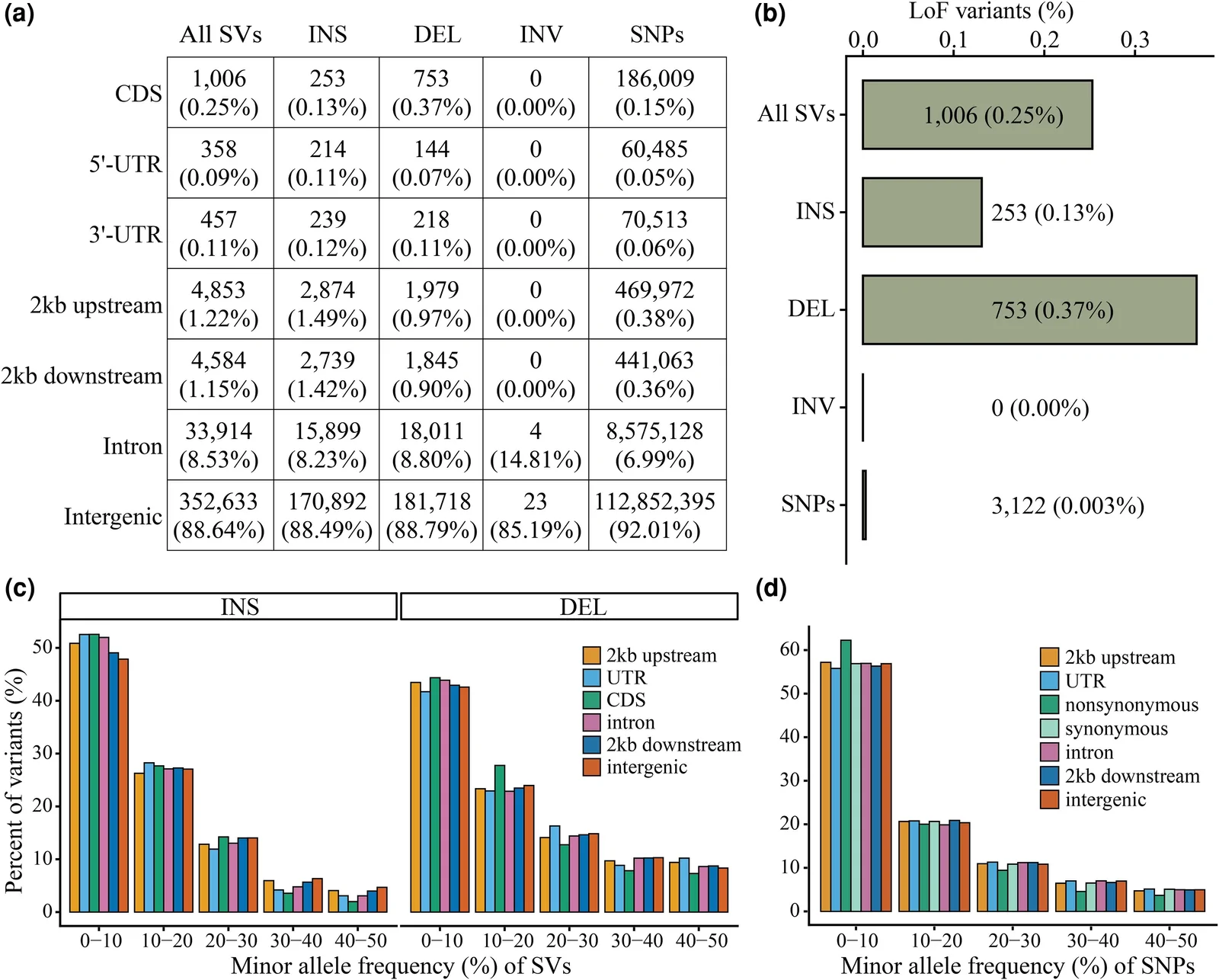
Graph-based SV genotyping in population Pd W and comparison with SNPs. a) Annotation of SVs and SNPs across genomic regions. Numbers indicate the counts and percentages of variants in each category. b) Proportion of LoF variants for SVs and SNPs. c and d) Minor allele frequency (MAF) spectra for SVs and SNPs overlapping different genomic features. The SV and SNP datasets were filtered for a missing rate <20%, a minor allele count (MAC) ≥1, and observed heterozygosity ≤50%.

### Signals of selection on SVs and SNPs

To compare the patterns of selection captured by SVs and SNPs in Pd W population, we applied local PCA using lostruct (Li and Ralph 2019) and selective sweep scans using SweepFinder2 (DeGiorgio et al. 2016) in nonoverlapping 1 Mb windows across the genome. Regions falling within the top 5% of both statistics were designated as candidate loci under selection in each dataset. By this criterion, the SV-based analysis identified candidate regions containing 4,401 SVs and 422 genes (Fig. 7a and Fig. S16; Supplementary Data S3), whereas the SNP-based analysis identified candidate regions containing 1,455,889 SNPs and 537 genes (Fig. 7b and Fig. S17; Supplementary Data S4). For completeness, all genes located within genomic regions falling in the top 5% of either the lostruct or SweepFinder2 method are provided in Supplementary Datas S5 to S8. Despite the large number of candidate genes detected in each dataset, only 155 genes were shared between the SV and SNP outliers (ie 19% of candidate gene loci in common), suggesting that the two marker types capture largely distinct signatures of selection.

**Figure 7:**
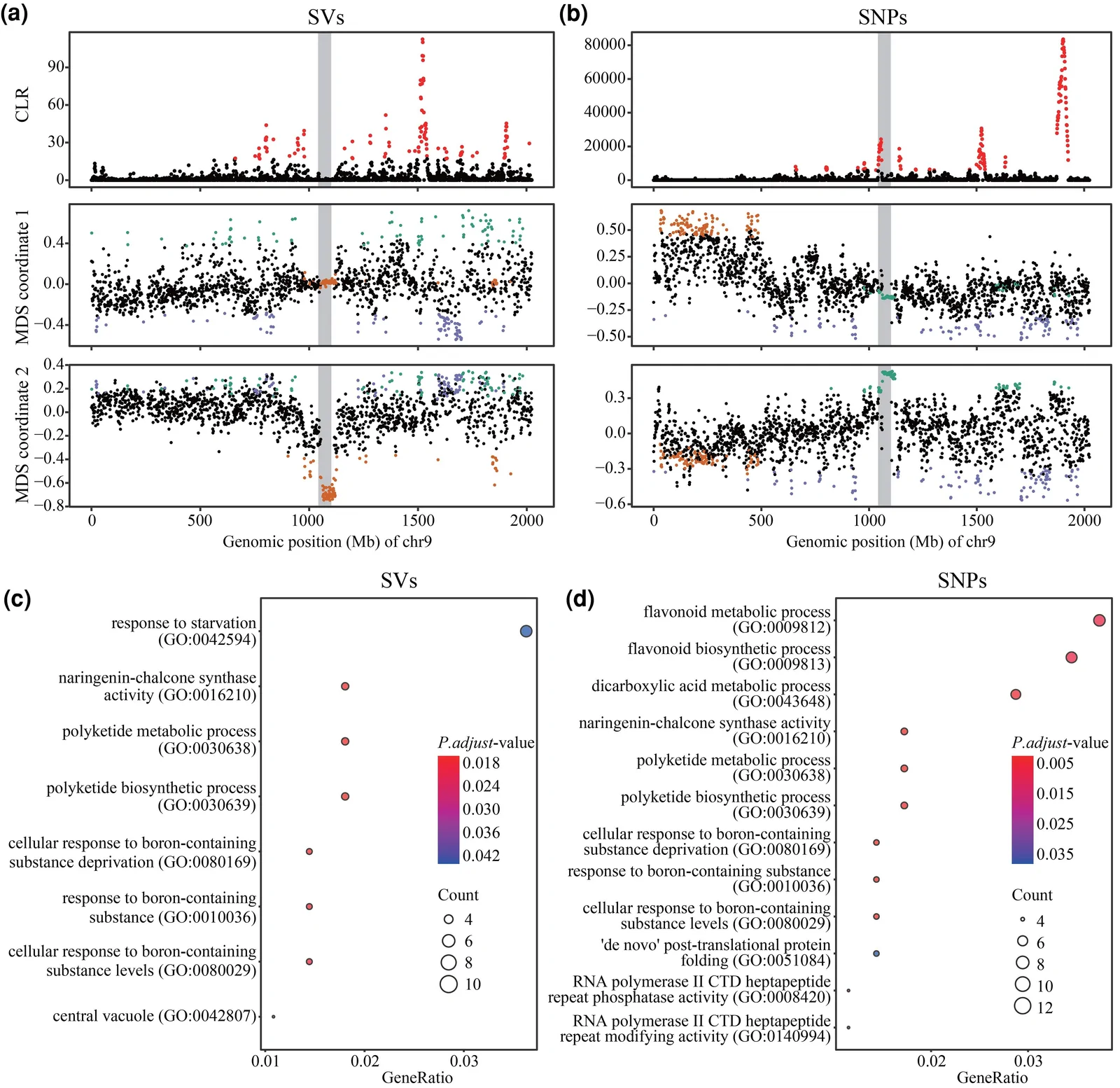
Genome scans for candidate regions under selection in population Pd W on chromosome 9. a and b) Dot plots of composite likelihood ratio (CLR) scores and multidimensional scaling (MDS) coordinates in the SV and SNP datasets. In CLR plots, red dots indicate windows in the genome-wide top 5% of CLR scores. In MDS plots, green, orange, and purple dots represent three sets of extreme windows located at the three corners of the two-dimensional MDS space, each set comprising 5% of all windows (see Figs. S16 and S17). Candidate regions under selection were defined as windows identified as outliers by both CLR and MDS analyses. The gray vertical bar indicates the putative centromeric region. Genome-wide results are shown in Figs. S16 and S17. c and d) GO enrichment of genes overlapping with SVs and SNPs outliers across the genome. GeneRatio indicates the proportion of genes assigned to each GO term. *P.adjust*-values are Benjamini–Hochberg (BH) adjusted *P*-value.

GO enrichment analyses revealed both shared and distinct functional signatures between SV and SNP outliers. Both showed significant enrichment of boron-response and secondary metabolite biosynthetic pathways (Fig. 7c and d). However, genes overlapping SV outliers were additionally enriched for categories related to vacuolar functions and nutrient deprivation, including central vacuole (GO:0042807) and response to starvation (GO:0042594) (Fig. 7c; Supplementary Data S9). On the other hand, genes overlapping SNP outliers were enriched for a broader range of metabolic and regulatory processes, including flavonoid biosynthetic process (GO:0009813), dicarboxylic acid metabolic process (GO:0043648), and de novo post-translational protein folding (GO:0051084) (Fig. 7d; Supplementary Data S10). These results suggest that SVs capture additional signatures of selection beyond those identified by SNPs, supporting their complementary roles in adaptation.

## Discussion

Understanding the full spectrum of genetic variation is fundamental to revealing the genetic basis of adaptive evolution. Yet structural variation, a major source of genomic diversity, remains largely unexplored in conifers because of technical challenges. This exploratory investigation provides a detailed characterization of structural variation in pines, establishes an integrated framework for graph-based SV genotyping, and assesses the opportunities and limitations of incorporating SVs into population genomic analyses of conifer trees.

### SVs in pine genome and association with TE activity

Advances in long-read sequencing have accelerated SV discovery in plant genomes, uncovering substantial variation in SV abundance among species (Table S8). In this study, we identified 5.7 million SVs across 21 pine individuals, with up to ∼1.5 million SVs per individual. Despite this extensive catalogue, the pan-SV accumulation curve remained unsaturated, suggesting that substantial structural variation remains undiscovered within the species complex. Consequently, the current SV catalogue and graph are likely to underestimate SV diversity when applied to more broadly sampled natural populations.

The exceptionally large number of SVs identified in pines reflects both their enormous genome size and their TE-rich genomic architecture. The pine genome, with a size of 20 to 30 Gb (Stevens et al. 2016; Niu et al. 2022; Jang et al. 2024; Neale et al. 2024), provides an extensive genomic substrate for the generation of structural variation. However, when scaled by genome size, structural diversity in pine, as measured by Watterson’s diversity estimator (κ_S_ = 5.22 × 10^−5^), is relatively low compared with other outcrossing perennial species. This suggests that the sheer number of SVs primarily reflects genome size rather than an unusually high per-base formation rate. In addition, the predominantly outcrossing and wind pollination mating system of pines promotes large effective population size (*N*e), increasing the efficacy of purifying selection against deleterious mutations and thereby shaping both the observed allele-frequency spectrum and functional composition of SVs.

TEs constitute ∼70% of the pine genome (Stevens et al. 2016; Niu et al. 2022; Jang et al. 2024; Neale et al. 2024) and are therefore expected to be a major driver of SV formation through mechanisms such as transposition, insertional mutagenesis, and non-allelic homologous recombination between repeated sequences (Kazazian 2004; Startek et al. 2015; Munasinghe et al. 2023). Consistent with this, the frequencies of TEs and SVs were positively correlated genome-wide. Furthermore, species- and population-specific SVs were more strongly associated with LTRs, tended to be shorter in length, and likely experienced faster turnover than shared SVs, indicative of more recent evolutionary origin. Together, these patterns highlight that TEs not only contribute substantially to the abundance and size distribution of SVs but also promote divergence among populations.

### Functional properties and evolutionary inferences

SVs can have diverse fitness effects depending on their type, size, and genomic context. Consistent with their greater potential to disrupt gene structure, SVs had a hundreds-of-times higher proportion of predicted LoF variants than SNPs, and LoF-associated SVs were longer than non-LoF SVs across all SV classes. Among the SV classes, TRAs constituted a large fraction of the LoF pool. Because TRA events involve chromosome breakage and rejoining at two distinct loci, they tend to be more disruptive to gene integrity (Weckselblatt and Rudd 2015). By contrast, INS and DEL, the most abundant SV types, showed relatively low proportions of predicted LoF effects.

Population-level SV frequency distributions further inform the evolutionary forces acting on this variant class. Findings from other systems suggest that SVs overlapping functionally important regions, such as CDS, should be enriched for rare alleles as a signature of purifying selection (Hämälä et al. 2021; Hämälä et al. 2024). To test this expectation while minimizing the confounding effects of sample size and population structure on the frequency spectrum, we examined graph-genotyped SVs within a single population (Pd W), using an expanded resequencing dataset of 11 individuals. Contrary to this prediction, however, SVs exhibited broadly similar MAF distributions across genomic contexts. SNPs genotyped in the same set of individuals, by contrast, showed the expected excess of rare nonsynonymous variants, consistent with ongoing selection against protein-altering mutations.

Several biological and technical factors may account for this discrepancy. First, the number of segregating CDS-overlapping SVs (1,006) was more than two orders of magnitude smaller than the number of nonsynonymous SNP sites (113,542), limiting the power to detect subtle shifts in the SV frequency spectrum. Second, the distribution of fitness effects may fundamentally differ between SVs and SNPs. Rather than spanning a continuum of effect sizes typical of missense mutations, SVs disrupting gene structure may have a more bimodal distribution of fitness effects, with variants being either largely neutral or strongly deleterious. Under this model, strongly deleterious SVs would be effectively purged by selection given the large *N*e of pine, leaving a highly filtered subset of mutational input with predominantly neutral or only mildly deleterious variants and thereby flattening the observed MAF spectrum. Third, functional annotation remains coarser for SVs than for SNPs. Even within each genomic context, SVs differ in size, breakpoint position, and predicted functional impact. This heterogeneity is likely to dilute category-level signatures of selection. Fourth, historical bottlenecks may have further contributed to the lack of detectable differences among SV categories by increasing the influence of genetic drift.

Technical limitations probably also contribute to the observed pattern. Although graph-based genotyping facilitates SV discovery from short-read data, many catalogued SVs could still not be confidently recovered (recall rate 44%), which likely affects rare and structurally complex variants disproportionately. This limitation is likely to bias population-level allele-frequency estimates and is an inherent challenge in repeat-rich conifer genomes. Disentangling biological processes from technical artifacts will require even larger population samples, refined functional annotation of SVs, and continued advances in graph-based genotyping, particularly for rare variants.

### SVs provide complementary insights into divergence and adaptation

Patterns and levels of population diversity and differentiation inferred from SVs and SNPs were broadly concordant, indicating that both marker classes primarily reflect the same underlying demographic history. This concordance is expected because the vast majority of both SVs and SNPs are intergenic, thus likely neutral or nearly neutral. Likewise, the retention of parental SVs in *P. densata* beyond random expectation is consistent with independent evidence of hybrid ancestry from SNP-based analyses (Wang et al. 2011; Zhao et al. 2020; Guo et al. 2023), further illustrating that SVs preserve the same broad demographic signal recovered from SNPs. These findings suggest that for reconstructing population structure, demographic history, and hybridization, SNP data alone may often be sufficient and more cost-effective to generate and analyze. However, SVs may provide additional resolution for fine-scale population structure under certain circumstances.

The picture changed, however, when we examined loci putatively involved in local adaptation. Selection scans revealed that only 19% of candidate genes identified by SVs and SNPs overlapped, the rest each capturing some unique functional categories, pointing to a coordinated physiological response to abiotic and biotic stress, including UV radiation, drought, extreme temperature, oxidative stress, and damage repairs (Xu et al. 2021; Li et al. 2025b). These functions are highly relevant to survival in the high-elevation environment of the Tibetan Plateau (Mao et al. 2009; Mao and Wang 2011; Li et al. 2025b). The limited overlap between marker classes suggests that SNPs and SVs capture partly distinct components of adaptive genetic variation rather than redundant signals. Integrating both marker classes therefore offers a more complete picture of genome evolution than either can provide alone.

Beyond their evolutionary significance, these findings have practical implications for tree breeding. One persistent challenge in conifer genomics is that SNP-based genome-wide association studies often explain only a small fraction of the heritable variation underlying complex traits (Chen et al. 2021; Hall et al. 2021; Bruxaux et al. 2024). Incorporating SV information into genomic selection models may help recover heritable variance currently unaccounted for by SNP-only approaches, potentially improving prediction accuracy for adaptive and breeding-relevant traits in conifer trees, as already demonstrated in crops (Liu et al. 2020; Zhou et al. 2022; Zhang et al. 2024a; Liu et al. 2024b; Yang et al. 2025).

### Challenges of SV detection in conifer gigagenomes

Our study highlights several inherent challenges in detecting and interpreting SVs in large, repeat-rich genomes. Paralogous sequences and active TE insertions cause multi-mapping and ambiguous read alignments, leading to missed or miscalled breakpoints, particularly for SVs embedded in repetitive elements. High genome-wide heterozygosity, typical of conifers, combined with low sequencing depth relative to genome size, further reduces confidence in heterozygous SV genotype calls. These factors may distort estimated allele frequencies and, by extension, any downstream inference drawn from the site frequency spectrum.

Few studies of structural variation report the criteria used to merge SV calls, making it difficult to assess the impact of this critical step on downstream analyses. We systematically evaluated 24 merging strategies that varied in SV sequence similarity, size similarity, and distance to breakpoint. Increasing the stringency of merging increased the proportion of low-frequency INS by ∼12%, illustrating the extent to which bioinformatic processing can bias estimates of SV diversity. Using a stringent calling strategy, we obtained a reasonably high-quality SV dataset supported by several independent lines of evidence: (i) consistency between SV- and SNP-based population structure and individual *H*o, (ii) depletion of SVs in CDS regions, and (iii) biologically consistent associations between SVs and TEs and between SV length and predicted LoF effects. These patterns are difficult to reconcile with a dataset dominated by random false positives and are therefore reassuring. On the other hand, graph-based SV genotyping using short reads achieved moderate performance with 44% recall and 70% precision (F1 = 0.54) relative to the long-reads derived benchmark catalogue. Although this performance is lower than that reported for *Arabidopsis thaliana*, rice, soya bean, and maize (Du et al. 2024), it is encouraging given the complexity of pine genomes and the predominance of INS and DEL in the SV catalogue, which are particularly challenging to genotype accurately from short reads. These results demonstrate both the promise and the current limitations of graph-based SV genotyping for large, repeat-rich genomes.

This study also shows that SVs arise and evolve via a variety of mechanisms that differ fundamentally from those underlying SNPs. Consequently, classical SNP-based evolutionary inferences, such as distribution of fitness effects of mutations, cannot necessarily be transferred directly to SV datasets because the underlying mutational processes and fitness consequences differ. Population genetic models built on SNP-specific assumptions are poorly suited to the diverse mutational mechanisms, wide size ranges, and recombinational consequences of SVs (Stuart et al. 2026). The field requires statistical frameworks tailored to SV evolution that explicitly account for these specificities. Progress will likely depend on continued improvements in long-read sequencing, pangenome-graph reference construction incorporating multiple high-quality assemblies per species, and larger population sample sizes to improve frequency-based inference. Such advances would allow more confident distinction between genuine evolutionary signal and technical artifacts of SV detection in these challenging genomic contexts.

## Materials and methods

### High molecular weight DNA isolation and long-read sequencing

To characterize SVs in pine genomes, we selected a set of 21 individuals from three closely related species for ONT long-read sequencing (Table S1). High molecular weight DNA was isolated from young seedlings following the protocol of nuclei isolation of the Nanobind PanDNA kit (PacBio, United States). Sequencing libraries were prepared using the Genomic DNA Ligation kit SQK-LSK109 or SQK-LSK110 (ONT, United Kingdom) following the manufacturer's procedure. Libraries were loaded onto R9.4.1 or R10.4.1 flow cells and sequenced using a PromethION platform (ONT, United Kingdom). Raw signal data stored in POD5 format was basecalled using the high-accuracy model in dorado v0.4.1 (https://github.com/nanoporetech/dorado), and the resulting FASTQ files were quality filtered by NanoFilt v2.8.0 (De Coster et al. 2018) to retain reads with a minimum length of 1,000 bp and an average quality score of at least 7 (“-l 1000 -q 7”).

### SV identification

To identify SVs, we aligned the filtered ONT long reads against the *P. tabuliformis* reference genome (Niu et al. 2022) using minimap2 v2.28 (Li 2018) with parameters “-x map-ont -a -Y -L --eqx --cs --MD --secondary=no”. The reference genome comprises a 25.4 Gb assembly with a contig N50 of 2.6 Mb and 96.1% of sequences anchored to 12 chromosomes (Niu et al. 2022). Reads with a mapping quality ≥20 were retained for SV calling. We first evaluated the performance of four SV-callers, including PBSV v2.10.0 (https://github.com/PacificBiosciences/pbsv), SVIM v2.0.0 (Heller and Vingron 2019), cuteSV v2.1.1 (Jiang et al. 2020), and Sniffles v2.4 (Smolka et al. 2024). Insertions and deletions were defined relative to the *P. tabuliformis* reference genome. SVs ranging from 50 bp to 1 Mb were scored. To ensure high confidence of SV calls in diploid genomes, the following filtering criteria were applied: (i) SVs are labeled as “PRECISE” by the callers; (ii) SVs have at least four supporting reads and coverage less than 2.5 × the genome average; (iii) SVs do not overlap with gaps in the reference genome. SVs meeting these criteria were merged across samples using Truvari v4.3.1 (English et al. 2022) with parameter settings --refdist 500 --pctseq 0 --pctsize 0, generating four SV datasets from the four callers. The performance of these methods appeared similar (see Results).

Long-read SV callers generally infer genotypes in diploids by comparing the numbers of reads supporting the reference and alternative alleles and assigning the genotype with the highest likelihood. For example, Sniffles uses a likelihood-based model in which the probability of observing SV-supporting reads is 0.05 for 0/0, 0.5 for 0/1, and 0.95 for 1/1 by default. In single-sample SV calling, only loci with evidence for an SV are typically reported in the output VCF. Consequently, loci that are truly homozygous reference (0/0) in a given sample are never explicitly genotyped. When callsets from multiple samples are subsequently merged, these loci appear as uncalled sites (./.) rather than as homozygous reference genotypes, leading to distorted genotype frequencies and biased estimates of population genetic diversity.

To generate an SV dataset suitable for both graph-pangenome construction and population-genetic analyses, we employed the multi-sample joint-calling framework implemented in Sniffles. The joint-calling first generates a unified catalogue of candidate SVs and then re-genotypes every sample at each candidate locus. Loci with sufficient read coverage but no SV-supporting reads can thereby be explicitly assigned as homozygous reference (0/0) genotypes rather than remaining uncalled. The benefit of joint-calling is that it converts a large proportion of previously uncalled sites into evidence-supported 0/0 calls, thereby reducing the number of missing genotypes in the final dataset.

Multiple Truvari merging parameter combinations (--refdist, --pctseq, and --pctsize) were subsequently evaluated to collapse redundant representations of the same biological SV and generate a nonredundant SV dataset. To assess the accuracy of SV calling, we manually inspected the long-read alignments of 20 randomly selected INS and DEL across five samples using IGV v2.19.4 (Thorvaldsdottir et al. 2013).

### Whole-genome re-sequencing and SNP calling

To evaluate the patterns of genetic diversity in SVs relative to SNPs, whole-genome re-sequencing was performed for 29 samples (Table S1) using an Illumina NovaSeq 6000 platform (by Benagen Technology Co., Ltd., Wuhan, China), generating 150 bp paired-end reads. Of these, 21 individuals corresponded to the ONT-sequenced samples used for SV discovery. Adapter sequences and low-quality bases (Phred quality < 20) were trimmed from both ends of each read using fastp v0.23.2 (Chen et al. 2018). The resulting clean reads were then mapped to the *P. tabuliformis* genome using BWA v.0.7.17 with default parameters (Li and Durbin 2009), generating BAM files. SNP calling was performed using BCFtools v1.19 (Li 2011). To minimize calling errors, several filtering steps were applied. Genotypes with genotype quality <20 or a read depth <4 were masked as missing. Additionally, loci were removed if they met any of the following criteria: missing rate >20%, mapping quality <40, number of alleles >2, or coverage depth exceeding 2.5× the genome-wide average.

### Graph-based SV genotyping

To construct a graph pangenome for population-scale genetic analyses, the *P. tabuliformis* reference genome and the identified SVs were integrated using the vg autoindex --workflow giraffe pipeline implemented in vg v1.73.0 (Garrison et al. 2018). Only DEL, INV, and sequence-resolved INS were included because vg does not support genotyping of DUP, TRA, or INS without resolved insertion sequences. A total of 29 Illumina-sequenced individuals (Table S1) were mapped to the graph pangenome using vg giraffe (Sirén et al. 2021). Read support across the graph was summarized using vg pack, with reads of mapping quality <5 and positions of base quality <5 excluded to filter low-confidence alignments. SV genotyping in the 29 individuals was then performed using vg call (Hickey et al. 2020). Additional filtering retained SVs supported by more than two reads, with read depth ≥4 and coverage depth <2.5× the average. Homozygous reference (0/0) and homozygous alternative (1/1) genotypes were required to be supported by more than two reads for the corresponding allele.

To assess the performance of graph-based SV genotyping, we compared the graph-based SV genotype calls with the ONT-derived SV callset for each of the 21 individuals with both ONT and Illumina sequencing data using Truvari. The ONT-derived SV callset served as the benchmark, and the graph-based SV callset as the comparison. Comparisons were performed separately for each individual in the benchmark and comparison callsets. Truvari was run with the parameters “--pctseq 0, --pctsize 0, --sizemin 50, --sizemax 1000000, --pick multi, and --no-ref a”. The --pick multi allows multiple benchmark variants to match a single comparison variant, whereas --no-ref a excludes homozygous reference (0/0) genotypes from the comparison. Precision, recall, and F1 score were calculated for each individual.

### Annotation of SNPs and SVs

To functionally annotate SVs and SNPs, we generated a set of high-quality genes from the *P. tabuliformis* reference by excluding incomplete genes, yielding a reference dataset of 49,715 genes. The functions of these genes were assigned using InterProScan v5.70-102.0 (Blum et al. 2021) and eggNOG-mapper v 2.1.12 (Cantalapiedra et al. 2021). SNPs were classified by genomic context (exonic, intronic, intergenic, and regulatory regions) and annotated using SnpEff v5.2c (Cingolani et al. 2012), with regulatory regions defined as the 2 kb up- and downstream of genes. LoF SNPs were defined based on SnpEff’s LoF annotations, which identify variants predicted to disrupt protein-coding genes through splice-site disruption, start codon loss, or premature stop codon gain.

SVs were assigned to genomic categories based on breakpoint overlap with genomic features identified using BEDTools v2.31.0 (Quinlan and Hall 2010). If an SV spans multiple regions, the assignment priority followed the order CDS, 5′ UTR, 3′ UTR, intron, 2 kb up- and downstream of genes, and intergenic. By this scheme, each SV was assigned to a single genomic category regardless of the number of breakpoints. Putative LoF SVs were defined following the SVAnnotate framework (https://gatk.broadinstitute.org/hc/en-us/articles/30332011989659-SVAnnotate). Specifically, DEL and INS overlapping CDS regions, INV disrupting CDS regions, DUP with both breakpoints in CDS regions or one breakpoint in CDS and the other in a UTR, and TRA with breakpoints within CDS or intron regions were all classified as LoF variants regardless of their lengths.

To assess the impact of repetitive sequences on SVs, we examined the overlap between SV breakpoint windows (±50 bp) and the repeat annotation of *P. tabuliformis* using BEDTools, requiring at least 60% of each breakpoint window to overlap an annotated repeat. Tandem repeats were identified using TRF v4.09 (Benson 1999), and TEs were annotated using EDTA (Extensive de-novo TE Annotator) v1.9.6 (Ou et al. 2019). Focusing on repeat identification surrounding the SV breakpoints rather than on the entire SV segment, which may contain numerous repeat sequences, especially in large SVs, is more useful for understanding the potential causal mechanisms of SV formation. Additionally, we analyzed the correlation between the distributions of SVs and TEs by screening 5 Mb nonoverlapping windows along the genome using BEDTools. The relationship was assessed by the Spearman correlation.

### Genetic diversity analyses

The SV and SNP datasets of 21 samples were used in a comparative population genetic analysis. Both SV and SNP datasets were filtered with a MAC ≥ 3 and a missing rate ≤20%. Individual *H*o across all loci and differentiation among sample groups (*F*_ST_) were calculated using VCFTOOLS v0.1.16 (Danecek et al. 2011). To perform PCA and population structure analysis, the variant datasets were filtered for linkage disequilibrium (LD; *r*^2^ ≤ 0.2) using PLINK v1.9b5 (Purcell et al. 2007). PCA was performed using PLINK, and population structure was inferred using ADMIXTURE v1.3.0 (Alexander et al. 2009), testing *K* values from 1 to 10 with 20 replicates for each *K*.

### Species (and population)-specific and species-shared SVs

To characterize structural divergence among species, we performed set intersection analyses using the isec function implemented in BCFtools. Presence–absence patterns of SVs among populations were visualized using UpSetR v1.4.0 (Conway et al. 2017), which provides an alternative to Venn diagrams for illustrating complex multi-set intersections.

To assess whether the SVs shared in *P. densata* with the two parental species (ie three-species-shared SVs) were by chance, we performed a permutation test that maintained the observed SV count in *P. densata* while randomly redistributing SV presence across loci. For each permutation, we counted the number of SVs shared among the species. Empirical *P*-values were calculated as (*k* + 1) / (*N* + 1), where *k* is the number of permutations yielding a test statistic equal to or more extreme than the observed value and *N* is the total number of permutations.

Genes overlapping these shared SVs were evaluated by GO enrichment analysis using clusterProfiler v4.14.0 (Wu et al. 2021). GO terms with Benjamini–Hochberg (BH)-adjusted *P*-values < 0.05 were considered significantly enriched. Because numerous GO terms were identified, we used Revigo v1.8.1 (Supek et al. 2011) to remove redundant terms and summarized the remaining terms into three categories: biological process, molecular function, and cellular component.

### Detection of selection in SV and SNP datasets

To identify genomic regions under selection, we applied lostruct (Li and Ralph 2019) and SweepFinder2 (DeGiorgio et al. 2016) on the genotyped SV and SNP datasets in population Pd W along nonoverlapping 1 Mb windows across the genome. Signals from the two detection methods were considered jointly; only windows in the top 5% across both methods were regarded as putative selected regions. Genes located within these regions were subjected to GO enrichment analysis using clusterProfiler. GO terms with Benjamini–Hochberg (BH)-adjusted *P* values < 0.05 were considered significantly enriched.

## Supplementary Material

msag239_Supplementary_Data

## Data Availability

Sequence data are archived in NCBI (PRJNA1444762) and National Genomics Data Center (NGDC) (https://ngdc.cncb.ac.cn/) (PRJCA049574). The SNP- and SV-call files are deposited in Zenodo (https://zenodo.org/uploads/20846370 and https://zenodo.org/uploads/20847076).
