# Supplementary material for: Uncovering structural variation in conifer gigagenomes: evolutionary insights and technical challenges
Source: Mol Biol Evol. 2026 Sep 18;43(10):msag239. doi: 10.1093/molbev/msag239 (PMC13637045; doi:10.1093/molbev/msag239)
Supplement: Supplementary file 1 [file msag239_supplementary_data.zip › Supplementary Figures and Tables final.pdf]

Uncovering structural variation in conifer gigagenomes: evolutionary insights and technical challenges

Hui Liu[1], Wei Zhao[1,3], Jing-Fang Guo[4], Xue-Mei Yan[5], Yan-Jing Liu[2], Qing-Yin Zeng[2], Xiao-Ru Wang[1]

[1]Department of Ecology, Environment and Geoscience, Umeå Plant Science Centre, Umeå University, Umeå, SE-90187, Sweden

[2]State Key Laboratory of Tree Genetics and Breeding, Chinese Academy of Forestry, Beijing 100091, China

[3]Laboratory of Tropical Forest Ecology, Xishuangbanna Tropical Botanical Garden, Chinese Academy of Sciences, Yunnan, Mengla 666303, China

[4]School of Horticulture and Food, Guangdong Eco-Engineering Polytechnic, Guangzhou, Guangdong 510520, China

[5]State Key Laboratory of Vegetable Biobreeding, Institute of Vegetables and Flowers, Chinese Academy of Agricultural Sciences, Beijing 100081, China

Supplemental information

Table S1. List of samples from *Pinus densata* (Pd), *P. tabuliformis* (Pt), and *P. yunnanensis* (Py) included in this study. *Pinus densata* samples were divided into eastern (Pd E), central (Pd C), and western (Pd W) groups. Sequencing platform, Oxford Nanopore Technologies (ONT) and/or Illumina, and data output for each individual, are provided with sequencing depth in parentheses.

Code	Sample ID	Species	Sequencing platform	Data output (Gb) ONT, Illumina	ONT N50 (kb)	Latitude (°N)	Longitude (°E)
1	HZPt01-02	Pt	ONT, Illumina	220 (8.7x), 308 (15.0x)	34	36.96	102.47
2	JZGPt01-03	Pt	ONT, Illumina	225 (8.9x), 258 (10.2x)	37	33.30	103.79
3	NCPt01-15	Pt	ONT, Illumina	224 (8.8x), 265 (10.4x)	30	42.28	118.97
4	TBPt01-01	Pt	ONT, Illumina	211 (8.3x), 748 (29.4x)	29	34.03	107.17
5	MKPd03-13	Pd E	ONT, Illumina	264 (10.4x), 339 (13.3x)	27	31.89	102.22
6	BXPd01-06	Pd E	ONT, Illumina	273 (10.7x), 357 (14.1x)	38	30.80	102.73
7	LXPd05-02	Pd E	ONT, Illumina	305 (12.0x), 449 (17.7x)	27	31.39	102.91
8	LXPd03-08	Pd E	ONT, Illumina	323 (12.7x), 321 (12.6x)	21	31.66	102.80
9	XCPd01-02	Pd C	ONT, Illumina	298 (11.7x), 523 (20.6x)	27	28.92	99.80
10	ZDPd01-18	Pd C	ONT, Illumina	307 (12.1x), 350 (13.8x)	40	28.44	98.87
11	ZDPd02-08	Pd C	ONT, Illumina	305 (12.0x), 269 (10.6x)	28	28.01	99.54
12	PdNX01-01	Pd C	ONT, Illumina	220 (8.7x), 275 (10.8x)	27	28.05	99.51
13	JLPd02-07	Pd C	ONT, Illumina	255 (10.0x), 321 (12.6x)	27	29.01	101.50
14	NYPd03-06	Pd W	ONT, Illumina	289 (11.4x), 401(15.8x)	33	29.88	93.65
15	NYPd03-02	Pd W	Illumina	468 (18.4x)	-	29.88	93.65
16	NYPd03-05	Pd W	Illumina	331 (13.0x)	-	29.88	93.65
17	PLPd02-09	Pd W	ONT, Illumina	292 (11.5x), 296 (11.7x)	30	29.90	95.50
18	PLPd02-05	Pd W	Illumina	266 (10.5x)	-	29.90	95.50
19	PLPd02-07	Pd W	Illumina	294 (11.6x)	-	29.90	95.50
20	PLPd02-11	Pd W	Illumina	299 (11.8x)	-	29.90	95.50
21	YLPd02-06	Pd W	ONT, Illumina	244 (9.6x), 356 (14.0x)	36	29.13	93.69
22	YLPd02-02	Pd W	Illumina	279 (11.0x)	-	29.13	93.69
23	YLPd02-10	Pd W	Illumina	268 (10.6x)	-	29.13	93.69
24	YLPd02-20	Pd W	Illumina	282 (11.1x)	-	29.13	93.69
25	YXPy01-08	Py	ONT, Illumina	264 (10.4x), 366 (14.4x)	36	24.26	102.16
26	BSPy01-10	Py	ONT, Illumina	223 (8.8x), 285 (11.2x)	35	24.48	99.14
27	LJPy01-07	Py	ONT, Illumina	239 (9.4x), 276 (10.9x)	35	26.89	100.24
28	YLPy01-06	Py	ONT, Illumina	218 (8.6x), 290 (11.4x)	38	24.72	103.17
29	GSPy02-04	Py	ONT, Illumina	264 (10.4x), 348 (13.7x)	26	25.97	98.82

Note: sequencing depth is calculated relative to *P. tabuliformis* genome 25.4 Gb.

Table S2. Summary of Oxford Nanopore Technologies (ONT) sequencing output and reads mapping rates for each individual. MAPQ, Mapping quality. Pt, *Pinus tabuliformis*; Pd, *P. densata*; Py, *P. yunnanensis*.

Sample ID	Species	ONT reads	Mapping rate	
			MAPQ > 0	MAPQ ≥ 20
HZPt01-02	Pt	14,050,861	99.84%	82.47%
JZGPt01-03	Pt	12,769,729	99.94%	83.10%
NCPt01-15	Pt	15,652,693	99.72%	82.27%
TBPt01-01	Pt	15,744,311	99.81%	81.18%
MKPd03-13	Pd E	23,582,378	99.89%	81.84%
BXPd01-06	Pd E	20,506,508	99.92%	82.30%
LXPd05-02	Pd E	30,443,514	99.85%	80.09%
LXPd03-08	Pd E	36,792,196	99.90%	81.79%
XCPd01-02	Pd C	28,206,478	99.45%	80.23%
ZDPd01-18	Pd C	20,586,647	99.92%	80.78%
ZDPd02-08	Pd C	29,058,488	99.88%	79.62%
PdNX01-01	Pd C	16,490,041	99.93%	78.35%
JLPd02-07	Pd C	24,023,962	99.74%	79.69%
NYPd03-06	Pd W	25,052,425	99.87%	80.14%
PLPd02-09	Pd W	26,115,447	99.96%	79.87%
YLPd02-06	Pd W	17,908,024	99.91%	79.85%
YXPy01-08	Py	23,886,995	99.91%	77.27%
BSPy01-10	Py	13,233,912	99.93%	79.13%
LJPy01-07	Py	13,515,109	99.89%	80.18%
YLPy01-06	Py	12,657,473	99.91%	79.77%
GSPy02-04	Py	24,097,153	99.95%	79.47%

Table S3. Non-redundant SVs identified by four callers in 21 ONT-sequenced samples and correlation of SVs with TEs. Non-redundant SVs were generated using merging parameters: --refdist 500 --pctsize 0.0 --pctseq 0.0.

Method	Number of SVs	Correlation with TEs (*P*-value)
Sniffles	4,876,355	0.46 (1.6E-253)
PBSV	4,976,967	0.45 (3.5E-237)
cuteSV	4,473,834	0.32 (8.4E-118)
SVIM	4,948,061	0.48 (2.2E-281)

Table S4. SV detection in 21 ONT-sequenced samples under different merging parameters using Truvari (English et al. 2022). Refdist, maximum breakpoint distance allowed for merging; pctsize, minimum allele size similarity required for merging; pctseq, minimum allele sequence similarity required for merging. INS, insertion; DEL, deletion; INV, inversion; DUP, duplication; TRA, translocation. The numbers indicate the counts of variants in each category with percentages in parentheses.

Merging parameters	All SVs	INS	DEL	INV	DUP	TRA
--refdist 500 --pctsize 0.0 --pctseq 0.0	4,500,476	2,310,971 (51.3)	1,879,366 (41.8)	6,758 (0.2)	26,677 (0.6)	276,704 (6.1)
--refdist 500 --pctsize 0.1 --pctseq 0.1	4,932,480	2,395,689 (48.6)	2,223,688 (45.1)	7,062 (0.1)	29,337 (0.6)	276,704 (5.6)
--refdist 500 --pctsize 0.2 --pctseq 0.2	5,024,662	2,430,090 (48.4)	2,281,340 (45.4)	7,191 (0.1)	29,337 (0.6)	276,704 (5.5)
--refdist 500 --pctsize 0.3 --pctseq 0.3	5,096,198	2,468,188 (48.4)	2,314,676 (45.4)	7,293 (0.1)	29,337 (0.6)	276,704 (5.4)
--refdist 500 --pctsize 0.4 --pctseq 0.4	5,159,840	2,506,466 (48.6)	2,339,944 (45.3)	7,389 (0.1)	29,337 (0.6)	276,704 (5.4)
--refdist 500 --pctsize 0.5 --pctseq 0.5	5,224,579	2,550,116 (48.8)	2,360,955 (45.2)	7,467 (0.1)	29,337 (0.6)	276,704 (5.3)
--refdist 500 --pctsize 0.6 --pctseq 0.6	5,295,322	2,602,249 (49.1)	2,379,457 (44.9)	7,575 (0.1)	29,337 (0.6)	276,704 (5.2)
--refdist 500 --pctsize 0.7 --pctseq 0.7	5,386,730	2,675,919 (49.7)	2,397,091 (44.5)	7,679 (0.1)	29,337 (0.5)	276,704 (5.1)
--refdist 500 --pctsize 0.8 --pctseq 0.8	5,551,940	2,812,100 (50.7)	2,425,920 (43.7)	7,879 (0.1)	29,337 (0.5)	276,704 (5.0)
--refdist 500 --pctsize 0.9 --pctseq 0.9	5,709,223	2,959,992 (51.8)	2,435,205 (42.7)	7,985 (0.1)	29,337 (0.5)	276,704 (4.8)
--refdist 500 --pctsize 1.0 --pctseq 1.0	5,850,388	3,096,540 (52.9)	2,439,652 (41.7)	8,155 (0.1)	29,337 (0.5)	276,704 (4.7)
--refdist 200 --pctsize 0.0 --pctseq 0.0	4,721,410	2,485,372 (52.6)	1,920,670 (40.7)	6,775 (0.1)	26,687 (0.6)	281,906 (6.0)
--refdist 100 --pctsize 0.0 --pctseq 0.0	4,811,887	2,559,056 (53.2)	1,934,963 (40.2)	6,784 (0.1)	26,692 (0.6)	284,392 (5.9)
--refdist 50 --pctsize 0.0 --pctseq 0.0	4,889,116	2,624,441 (53.7)	1,944,575 (39.8)	6,788 (0.1)	26,695 (0.5)	286,617 (5.9)
--refdist 50 --pctsize 0.1 --pctseq 0.1	5,235,092	2,644,027 (50.5)	2,268,028 (43.3)	7,083 (0.1)	29,337 (0.6)	286,617 (5.5)
--refdist 50 --pctsize 0.2 --pctseq 0.2	5,291,355	2,654,406 (50.2)	2,313,787 (43.7)	7,208 (0.1)	29,337 (0.6)	286,617 (5.4)
--refdist 50 --pctsize 0.3 --pctseq 0.3	5,331,259	2,667,615 (50.0)	2,340,384 (43.9)	7,306 (0.1)	29,337 (0.6)	286,617 (5.4)
--refdist 50 --pctsize 0.4 --pctseq 0.4	5,367,677	2,683,587 (50.0)	2,360,737 (44.0)	7,399 (0.1)	29,337 (0.5)	286,617 (5.3)
--refdist 50 --pctsize 0.5 --pctseq 0.5	5,406,780	2,705,084 (50.0)	2,378,266 (44.0)	7,476 (0.1)	29,337 (0.5)	286,617 (5.3)
--refdist 50 --pctsize 0.6 --pctseq 0.6	5,450,633	2,733,333 (50.1)	2,393,766 (43.9)	7,580 (0.1)	29,337 (0.5)	286,617 (5.3)
--refdist 50 --pctsize 0.7 --pctseq 0.7	5,502,114	2,770,831 (50.4)	2,407,647 (43.8)	7,682 (0.1)	29,337 (0.5)	286,617 (5.2)
--refdist 50 --pctsize 0.8 --pctseq 0.8	5,588,329	2,838,239 (50.8)	2,426,257 (43.4)	7,879 (0.1)	29,337 (0.5)	286,617 (5.1)
--refdist 50 --pctsize 0.9 --pctseq 0.9	5,730,399	2,971,104 (51.8)	2,435,356 (42.5)	7,985 (0.1)	29,337 (0.5)	286,617 (5.0)
--refdist 50 --pctsize 1.0 --pctseq 1.0	5,860,304	3,096,543 (52.8)	2,439,652 (41.6)	8,155 (0.1)	29,337 (0.5)	286,617 (4.9)

Table S5. Effect of merging strategies on detection of low-frequency SV alleles (minor allele frequency ≤ 5%) in 21 ONT-sequenced individuals. Refdist, maximum breakpoint distance allowed for merging; pctsize, minimum allele size similarity required for merging; pctseq, minimum allele sequence similarity required for merging. INS, insertion; DEL, deletion; INV, inversion; DUP, duplication; TRA, translocation.

Merging parameters	INS (%)	DEL (%)	INV (%)	DUP (%)	TRA (%)
--refdist 500 --pctsize 0.0 --pctseq 0.0	34.94	31.58	50.86	62.37	64.53
--refdist 500 --pctsize 0.1 --pctseq 0.1	36.17	34.33	51.95	65.70	64.53
--refdist 500 --pctsize 0.2 --pctseq 0.2	36.73	34.99	52.18	65.70	64.53
--refdist 500 --pctsize 0.3 --pctseq 0.3	37.37	35.34	52.40	65.70	64.53
--refdist 500 --pctsize 0.4 --pctseq 0.4	38.00	35.61	52.63	65.70	64.53
--refdist 500 --pctsize 0.5 --pctseq 0.5	38.69	35.84	52.66	65.70	64.53
--refdist 500 --pctsize 0.6 --pctseq 0.6	39.51	36.06	52.99	65.70	64.53
--refdist 500 --pctsize 0.7 --pctseq 0.7	40.67	36.26	53.16	65.70	64.53
--refdist 500 --pctsize 0.8 --pctseq 0.8	42.99	36.58	53.53	65.70	64.53
--refdist 500 --pctsize 0.9 --pctseq 0.9	45.25	36.71	53.55	65.70	64.53
--refdist 500 --pctsize 1.0 --pctseq 1.0	46.65	36.76	53.91	65.70	64.53
--refdist 200 --pctsize 0.0 --pctseq 0.0	37.71	32.07	50.93	62.39	65.40
--refdist 100 --pctsize 0.0 --pctseq 0.0	39.05	32.23	50.98	62.40	65.76
--refdist 50 --pctsize 0.0 --pctseq 0.0	40.27	32.33	50.96	62.41	66.08
--refdist 50 --pctsize 0.1 --pctseq 0.1	40.52	34.71	52.03	65.70	66.08
--refdist 50 --pctsize 0.2 --pctseq 0.2	40.67	35.24	52.25	65.70	66.08
--refdist 50 --pctsize 0.3 --pctseq 0.3	40.87	35.54	52.45	65.70	66.08
--refdist 50 --pctsize 0.4 --pctseq 0.4	41.12	35.78	52.66	65.70	66.08
--refdist 50 --pctsize 0.5 --pctseq 0.5	41.44	35.98	52.71	65.70	66.08
--refdist 50 --pctsize 0.6 --pctseq 0.6	41.85	36.18	53.02	65.70	66.08
--refdist 50 --pctsize 0.7 --pctseq 0.7	42.37	36.35	53.17	65.70	66.08
--refdist 50 --pctsize 0.8 --pctseq 0.8	43.43	36.58	53.53	65.70	66.08
--refdist 50 --pctsize 0.9 --pctseq 0.9	45.41	36.71	53.55	65.70	66.08
--refdist 50 --pctsize 1.0 --pctseq 1.0	46.65	36.76	53.91	65.70	66.08

Table S6. Summary of SVs detected in 21 ONT-sequenced individuals under merging parameters: --refdist 500 --pctsize 0.9 --pctseq 0.9. Pt, *Pinus tabuliformis*; Pd, *P. densata*; Py, *P. yunnanensis*.

Sample ID	Species	INS	DEL	INV	DUP	TRA	All SVs
HZPt01-02	Pt	375,231	486,273	1,065	2,273	12,156	876,998
JZGPt01-03	Pt	391,335	506,689	1,118	2,347	12,500	913,989
NCPt01-15	Pt	361,018	476,134	1,094	2,447	12,354	853,047
TBPt01-01	Pt	348,290	457,462	1,033	2,242	10,996	820,023
MKPd03-13	Pd E	537,432	652,074	1,520	3,655	25,295	1,219,976
BXPd01-06	Pd E	584,801	693,823	1,487	3,349	24,303	1,307,763
LXPd05-02	Pd E	445,427	585,468	1,420	3,441	25,736	1,061,492
LXPd03-08	Pd E	538,627	667,750	1,576	3,891	28,514	1,240,358
XCPd01-02	Pd C	621,446	731,989	1,764	4,639	39,480	1,399,318
ZDPd01-18	Pd C	663,420	765,596	1,710	4,072	36,046	1,470,844
ZDPd02-08	Pd C	624,486	735,153	1,748	4,610	38,854	1,404,851
PdNX01-01	Pd C	435,046	547,958	1,272	3,293	22,495	1,010,064
JLPd02-07	Pd C	557,072	663,141	1,487	4,102	29,377	1,255,179
NYPd03-06	Pd W	616,260	710,552	1,633	4,189	38,980	1,371,614
PLPd02-09	Pd W	608,662	707,160	1,670	4,323	39,204	1,361,019
YLPd02-06	Pd W	565,428	654,566	1,484	3,554	27,965	1,252,997
YXPy01-08	Py	587,399	694,256	1,528	3,545	26,647	1,313,375
BSPy01-10	Py	487,195	600,292	1,323	3,004	20,438	1,112,252
LJPy01-07	Py	508,795	622,055	1,372	3,269	23,667	1,159,158
YLPy01-06	Py	482,840	592,432	1,334	2,916	18,683	1,098,205
GSPy02-04	Py	562,265	676,649	1,562	3,962	31,289	1,275,727

Table S7. Manual validation in Integrative Genomics Viewer (IGV) for 20 selected INS and DEL variants (--refdist 500 --pctsize 0.9 --pctseq 0.9 SV set) across five samples. Because of the large size of the reference genome chromosomes, each chromosome (chr1–chr12) was partitioned into smaller segments, resulting in 56 segments (ID: Chr1–Chr56) for bioinformatic analyses. Each SV identifier consists of the SV type, chromosome segment ID, and start coordinate within the corresponding segment. “Yes" means that the called genotype was verified by IGV, with examples shown in Figure S3.

Chr	Position	SV ID	Length (bp)	HZPt01-02	MKPd03-13	XCPd01-02	NYPd03-06	YXPy01-08
chr1	1,113,606,721	INS_Chr3_113606721	1,217	0/0 (Yes)	0/1 (No)	0/1 (Yes)	1/1 (Yes)	1/1 (Yes)
chr2	768,216,025	INS_Chr7_268216025	69	1/1 (No)	0/0 (Yes)	0/0 (Yes)	1/1 (No)	1/1 (Yes)
chr3	951,163,832	INS_Chr12_451163832	176	0/1 (Yes)	./. (Yes)	0/1 (Yes)	0/0 (Yes)	0/1 (Yes)
chr5	1,692,274,960	INS_Chr24_192274960	77	0/1 (Yes)	0/0 (Yes)	0/0 (Yes)	0/1 (Yes)	0/1 (Yes)
chr6	603,106,210	INS_Chr27_103106210	288	0/1 (Yes)	0/0 (Yes)	0/0 (Yes)	0/1 (Yes)	1/1 (Yes)
chr7	1,658,908,569	INS_Chr34_158908569	131	0/1 (No)	0/1 (Yes)	1/1 (No)	0/0 (Yes)	0/0 (Yes)
chr8	330,765,542	INS_Chr36_330765542	91	0/0 (Yes)	0/1 (No)	1/1 (Yes)	1/1 (Yes)	0/1 (No)
chr9	461,804,817	INS_Chr41_461804817	58	0/0 (Yes)	0/1 (Yes)	1/1 (Yes)	1/1 (Yes)	1/1 (Yes)
chr10	151,684,006	INS_Chr46_151684006	124	0/0 (Yes)	0/0 (Yes)	1/1 (Yes)	0/0 (Yes)	1/1 (Yes)
chr11	151,162,563	INS_Chr50_151162563	245	0/0 (Yes)	0/0 (Yes)	1/1 (Yes)	0/0 (Yes)	1/1 (Yes)
chr2	607,400,461	DEL_Chr7_107400461	650	1/1 (Yes)	0/1 (Yes)	0/0 (Yes)	0/1 (Yes)	1/1 (Yes)
chr3	773,656,955	DEL_Chr12_273656955	214	0/1 (Yes)	0/1 (Yes)	0/1 (Yes)	0/0 (Yes)	0/1 (No)
chr4	688,032,942	DEL_Chr17_188032942	179	0/0 (Yes)	./. (Yes)	0/1 (No)	1/1 (Yes)	1/1 (Yes)
chr5	850,935,845	DEL_Chr22_350935845	276	0/0 (Yes)	0/0 (Yes)	1/1 (Yes)	1/1 (Yes)	0/1 (No)
chr6	687,255,177	DEL_Chr27_187255177	7,632	0/0 (Yes)	1/1 (Yes)	0/1 (No)	0/0 (Yes)	0/0 (Yes)
chr7	364,581,017	DEL_Chr31_364581017	66	0/0 (Yes)	0/0 (Yes)	1/1 (Yes)	0/1 (Yes)	0/0 (Yes)
chr8	1,371,379,410	DEL_Chr38_371379410	670	0/0 (Yes)	0/1 (Yes)	1/1 (Yes)	1/1 (Yes)	0/1 (No)
chr9	443,256,078	DEL_Chr41_443256078	82	0/0 (Yes)	0/1 (Yes)	1/1 (Yes)	1/1 (Yes)	1/1 (Yes)
chr11	384,131,281	DEL_Chr50_384131281	67	0/0 (Yes)	0/1 (Yes)	1/1 (Yes)	0/0 (Yes)	1/1 (Yes)
chr12	1,261,631,590	DEL_Chr56_261631590	67	0/1 (Yes)	1/1 (Yes)	0/0 (Yes)	0/1 (Yes)	0/1 (Yes)

Table S8. Summary of datasets used to calculate Watterson's structural diversity estimate (κ_S) in plant species. In the Life cycle category, biennial species were grouped with annual species. In the Mating system category, species classified as having a mixed mating system were not included in the plot shown in Figure 1.

Species	Life cycle	Mating system	# Samples	# SVs	κ_S	Reference
Arabidopsis thaliana	Annual	Selfing	32	61,322	9.68E-05	(Kang et al. 2023)
Brassica napus	Annual/Biennial	Mixed	57	94,824	1.71E-05	(Yildiz et al. 2025)
Brassica rapa	Annual/Biennial	Outcrossing	30	276,700	0.000137	(Ma et al. 2026)
Camellia sinensis	Perennial	Outcrossing	22	866,903	6.51E-05	(Chen, Wang, et al. 2023)
Citrullus	Annual	Outcrossing	137	910,844	0.000398	(Sun et al. 2026)
Cucumis melo	Annual	Mixed	25	108,000	6.74E-05	(Oren et al. 2022)
Glycine max	Annual	Selfing	26	124,222	2.72E-05	(Liu et al. 2020)
Gossypium barbadense	Perennial	Mixed	16	129,673	1.22E-05	(Meng et al. 2025)
Hordeum vulgare	Annual	Selfing	19	1,586,262	8.12E-05	(Jayakodi et al. 2020)
Malus	Perennial	Outcrossing	36	472,587	0.000142	(Li et al. 2025)
Oryza	Annual	Selfing	251	193,880	7.43E-05	(Shang et al. 2022)
Oryza sativa	Annual	Selfing	31	171,072	9.48E-05	(Qin et al. 2021)
Panicum miliaceum	Annual	Selfing	32	50,689	1.11E-05	(Chen, Liu, et al. 2023)
Populus	Perennial	Outcrossing	19	142,202	8.63E-05	(Shi et al. 2024)
Prunus mume	Perennial	Outcrossing	18	51,461	5.33E-05	(Huang et al. 2026)
Quercus variabilis	Perennial	Outcrossing	22	543,372	0.000159	(Liang et al. 2025)
Rosa	Perennial	Outcrossing	26	1,801,537	0.000731	(Zhang et al. 2026)
Setaria	Annual	Selfing	112	202,884	8.45E-05	(He et al. 2023)
Solanum lycopersicum	Annual	Selfing	32	195,957	5.17E-05	(Zhou et al. 2022)
Solanum melongena	Annual/Biennial	Selfing	15	76,481	1.65E-05	(You et al. 2026)
Solanum Section *Petota*	Perennial	Mixed	44	561,433	0.00015	(Tang et al. 2022)
Theobroma cacao	Perennial	Mixed	31	36,303	2.52E-05	(Hämälä et al. 2021)
Triticum aestivum	Annual	Selfing	17	249,976	3.30E-06	(Jiao et al. 2025)
Vitis	Perennial	Outcrossing	29	236,449	0.000103	(Liu et al. 2024)
Zea mays	Annual	Outcrossing	55	2,969,080	0.000258	(Yang et al. 2025)
Pinus	Perennial	Outcrossing	21	5,709,223	5.22E-05	This study

Table S9. Summary of graph-based SV genotyping in 29 Illumina-sequenced individuals. Of these samples, 21 (marked in bold) were sequenced using both ONT and Illumina. Pt, *Pinus tabuliformis*; Pd, *P. densata*; Py, *P. yunnanensis*.

Sample ID	Species	Properly paired reads in pangenome (%; MAPQ > 0)	INS	DEL	INV	All SVs
**HZPt01-02**	Pt	97.40	188,939	367,185	29	556,153
**JZGPt01-03**	Pt	97.69	173,130	345,988	19	519,137
**NCPt01-15**	Pt	97.84	168,451	341,092	18	509,561
**TBPt01-01**	Pt	97.00	241,553	444,905	42	686,500
**MKPd03-13**	Pd E	96.39	267,696	411,383	19	679,098
**BXPd01-06**	Pd E	97.45	289,947	442,647	29	732,623
**LXPd05-02**	Pd E	97.19	212,921	372,659	30	585,610
**LXPd03-08**	Pd E	97.39	236,145	398,253	21	634,419
**XCPd01-02**	Pd C	97.51	394,626	514,278	52	908,956
**ZDPd01-18**	Pd C	97.52	333,635	442,156	34	775,825
**ZDPd02-08**	Pd C	97.47	305,015	422,743	24	727,782
**PdNX01-01**	Pd C	97.56	256,720	381,803	29	638,552
**JLPd02-07**	Pd C	97.42	313,494	435,300	25	748,819
**NYPd03-06**	Pd W	97.33	357,725	440,718	34	798,477
NYPd03-02	Pd W	97.13	359,608	450,491	34	810,133
NYPd03-05	Pd W	97.10	306,530	400,036	31	706,597
**PLPd02-09**	Pd W	97.37	312,229	396,627	33	708,889
PLPd02-05	Pd W	97.45	307,175	396,016	23	703,214
PLPd02-07	Pd W	97.54	331,317	420,474	27	751,818
PLPd02-11	Pd W	97.41	324,196	411,985	24	736,205
**YLPd02-06**	Pd W	97.40	339,754	424,730	34	764,518
YLPd02-02	Pd W	97.13	312,083	398,686	31	710,800
YLPd02-10	Pd W	97.54	293,427	380,128	22	673,577
YLPd02-20	Pd W	97.47	298,532	386,657	22	685,211
**YXPy01-08**	Py	97.35	339,134	462,974	43	802,151
**BSPy01-10**	Py	97.36	286,321	426,133	25	712,479
**LJPy01-07**	Py	97.73	278,735	411,809	24	690,568
**YLPy01-06**	Py	97.37	284,531	419,515	27	704,073
**GSPy02-04**	Py	97.54	338,344	461,238	39	799,621

Table S10. Truvari benchmarking results (recall, precision and F1 score) of graph-based SV genotyping in 21 Illumina-sequenced samples against long-reads derived SV calls in the same set of samples. The F1 score was calculated as the harmonic mean of recall and precision.

Sample ID	Species	INS			DEL			INV			All SVs		
		Recall	Precision	F1	Recall	Precision	F1	Recall	Precision	F1	Recall	Precision	F1
HZPt01-02	Pt	0.29	0.56	0.38	0.49	0.64	0.55	0.02	0.79	0.04	0.40	0.61	0.48
JZGPt01-03	Pt	0.26	0.58	0.36	0.46	0.65	0.54	0.01	0.79	0.03	0.37	0.63	0.47
NCPt01-15	Pt	0.27	0.56	0.36	0.47	0.64	0.54	0.01	0.78	0.03	0.38	0.61	0.47
TBPt01-01	Pt	0.34	0.49	0.40	0.57	0.58	0.58	0.03	0.62	0.05	0.47	0.55	0.51
MKPd03-13	Pd E	0.34	0.66	0.44	0.48	0.73	0.58	0.01	0.84	0.02	0.41	0.70	0.52
BXPd01-06	Pd E	0.36	0.71	0.48	0.50	0.76	0.60	0.02	0.79	0.03	0.44	0.74	0.55
LXPd05-02	Pd E	0.34	0.69	0.45	0.51	0.77	0.61	0.02	0.97	0.04	0.43	0.74	0.55
LXPd03-08	Pd E	0.34	0.75	0.46	0.49	0.78	0.60	0.01	0.90	0.02	0.42	0.77	0.54
XCPd01-02	Pd C	0.45	0.70	0.55	0.57	0.79	0.66	0.02	0.79	0.05	0.52	0.75	0.61
ZDPd01-18	Pd C	0.39	0.75	0.51	0.49	0.81	0.61	0.01	0.59	0.02	0.44	0.78	0.56
ZDPd02-08	Pd C	0.36	0.73	0.49	0.48	0.80	0.60	0.01	0.71	0.02	0.43	0.77	0.55
PdNX01-01	Pd C	0.35	0.59	0.44	0.50	0.70	0.58	0.02	0.69	0.03	0.43	0.65	0.52
JLPd02-07	Pd C	0.38	0.67	0.49	0.51	0.75	0.60	0.01	0.76	0.03	0.45	0.71	0.55
NYPd03-06	Pd W	0.42	0.75	0.54	0.52	0.81	0.63	0.02	0.88	0.04	0.47	0.78	0.59
PLPd02-09	Pd W	0.38	0.75	0.50	0.47	0.81	0.60	0.01	0.76	0.03	0.43	0.79	0.55
YLPd02-06	Pd W	0.41	0.71	0.52	0.51	0.77	0.61	0.02	0.82	0.04	0.46	0.74	0.57
YXPy01-08	Py	0.41	0.70	0.52	0.53	0.76	0.62	0.02	0.74	0.04	0.47	0.74	0.58
BSPy01-10	Py	0.36	0.60	0.45	0.51	0.71	0.59	0.01	0.72	0.03	0.44	0.66	0.53
LJPy01-07	Py	0.35	0.62	0.44	0.49	0.73	0.59	0.01	0.79	0.03	0.43	0.68	0.52
YLPy01-06	Py	0.36	0.60	0.45	0.50	0.70	0.59	0.01	0.70	0.03	0.44	0.66	0.53
GSPy02-04	Py	0.41	0.67	0.51	0.53	0.76	0.62	0.02	0.72	0.03	0.48	0.72	0.57
Average	-	0.36	0.66	0.46	0.50	0.73	0.60	0.02	0.77	0.03	0.44	0.70	0.54

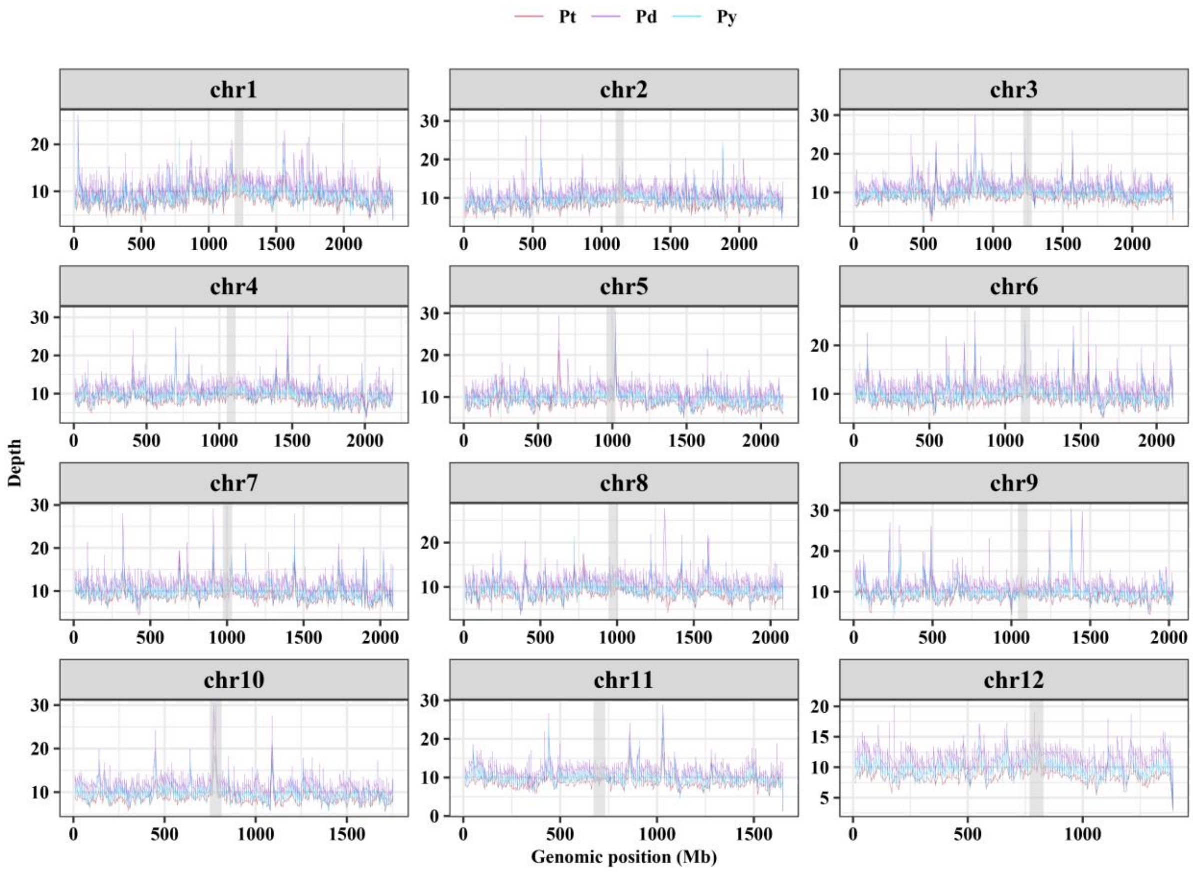

Figure S1. Mapping depth of the 21 ONT-sequenced samples of *P. densata* (Pd), *P. tabuliformis* (Pt), and *P. yunnanensis* (Py) along 12 chromosomes of the reference genome. The grey shaded regions indicate putative centromeric regions. Mapping depth was calculated across 10-Mb non-overlapping genomic windows.

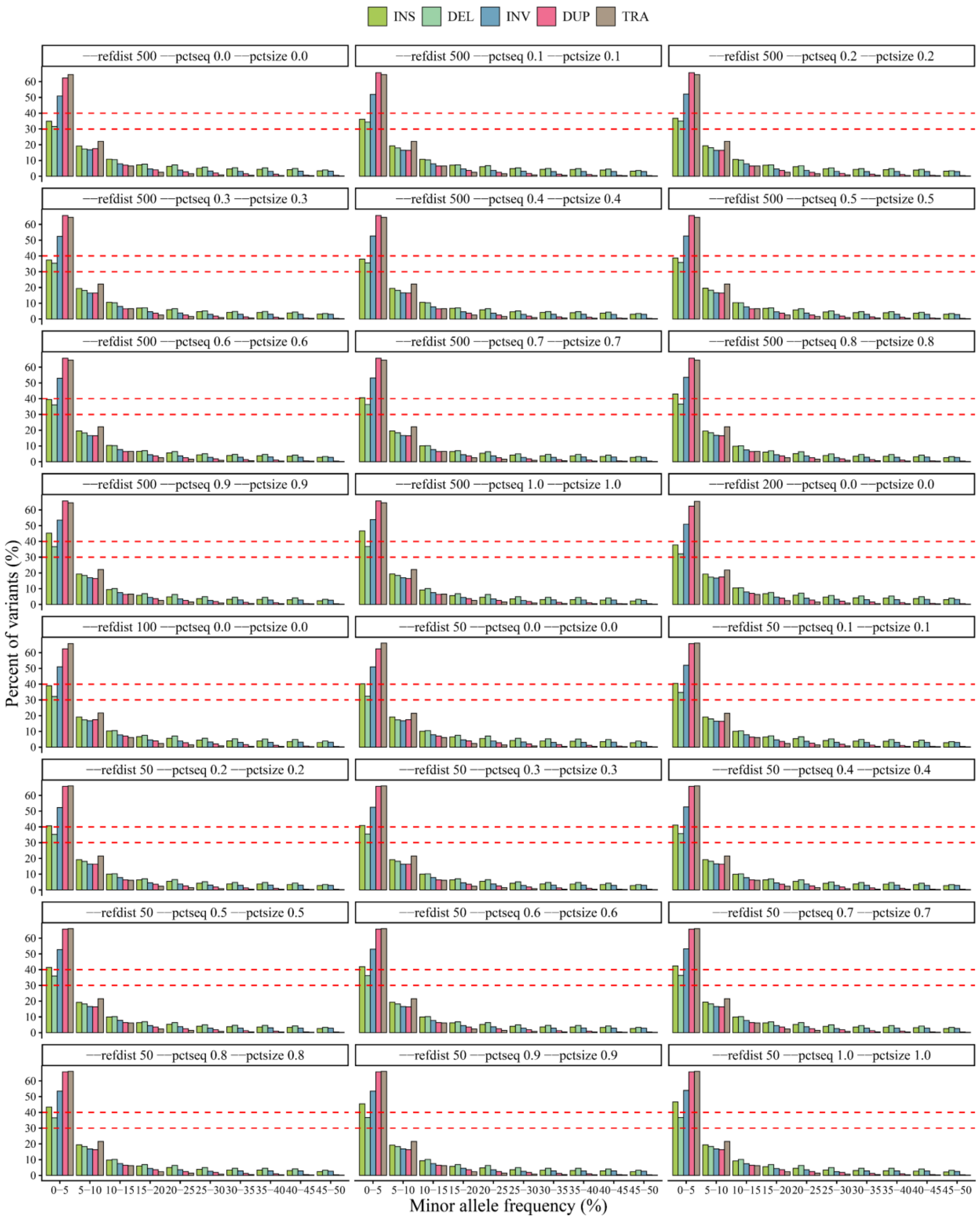

Figure S2. Minor allele frequency (MAF) spectra of SVs detected in 21 ONT-sequenced samples under different merging parameters. Red dashed lines indicate 30% and 40%.

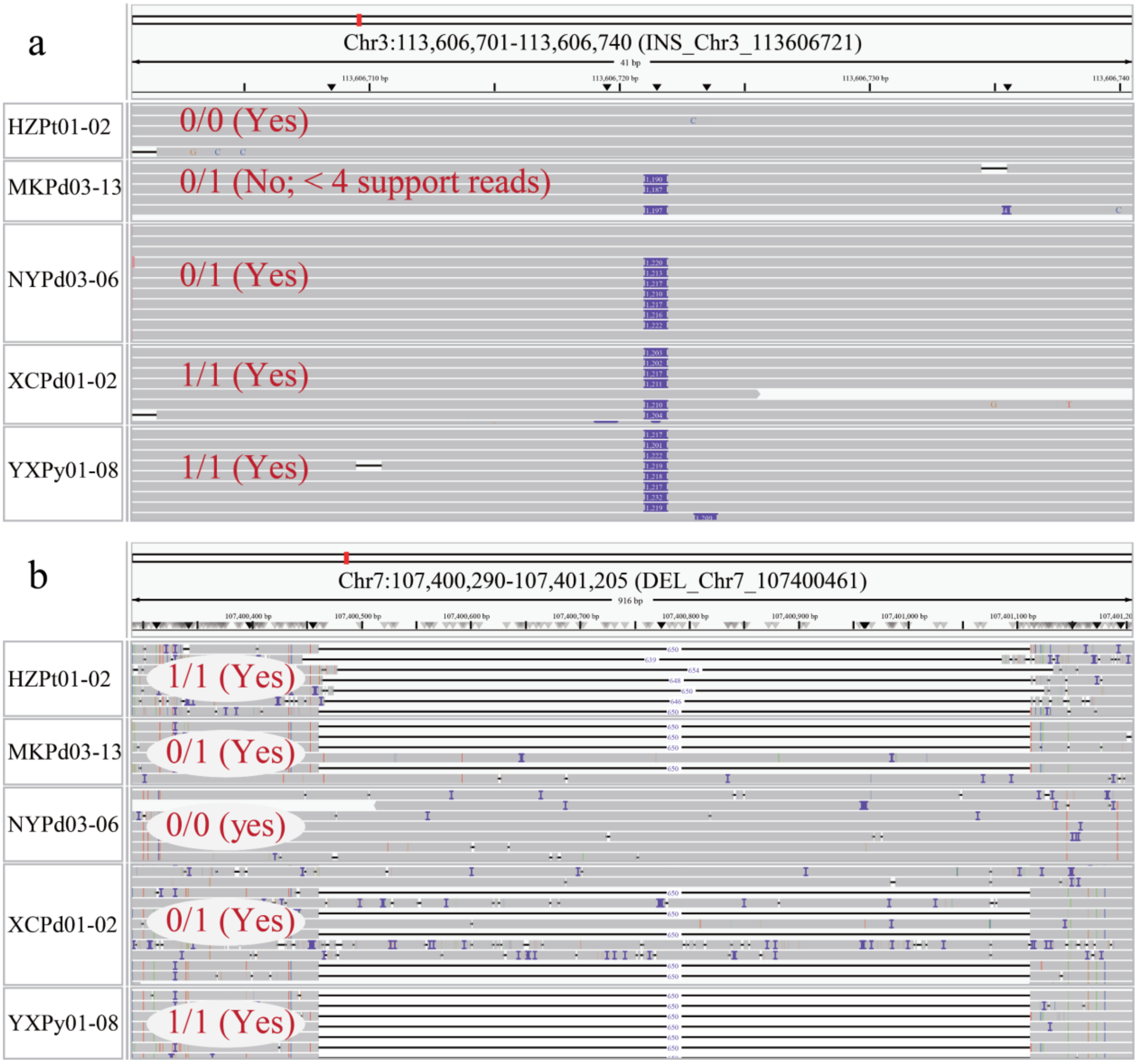

Figure S3. Examples of manual validation of insertions (a) and deletions (b) in five samples using long-read alignments to the reference genome in Integrative Genomics Viewer (see also Table S7). Because of the large size of the reference genome chromosomes, each chromosome (chr1–chr12) was partitioned into smaller segments, resulting in 56 segments (ID: Chr1–Chr56) for bioinformatic analyses. Each SV identifier specifies the SV type, chromosome segment ID and start coordinate within the segment.

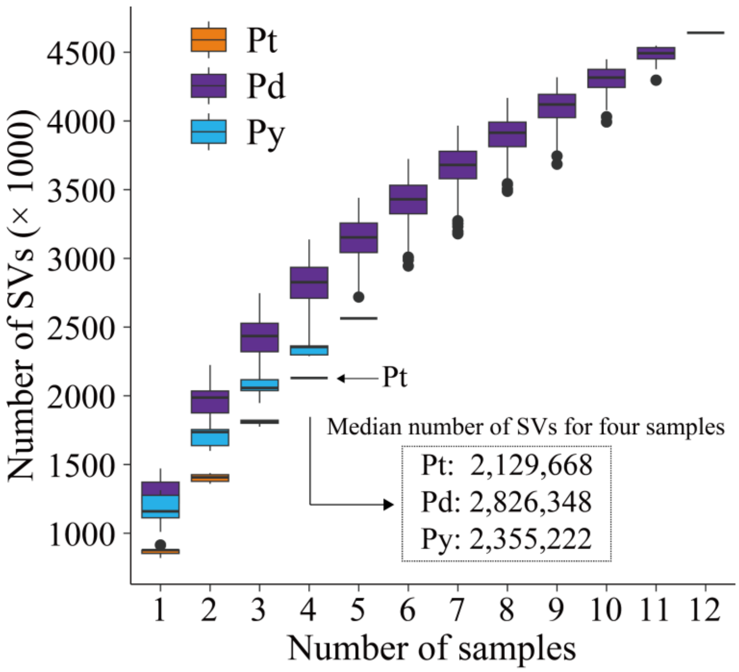

Figure S4. Accumulation curves of pan-SVs with increasing sample size in the three pine species. Order of samples being added into the pan-SVs with 1,000 bootstraps. Values in the dashed box indicate the median number of SVs at a rarefied sample size of four individuals per species. At a sample size of four individuals, all Pt samples were included in every bootstrap replicate, resulting in identical SV counts across replicates and causing the boxplot to collapse to a single line.

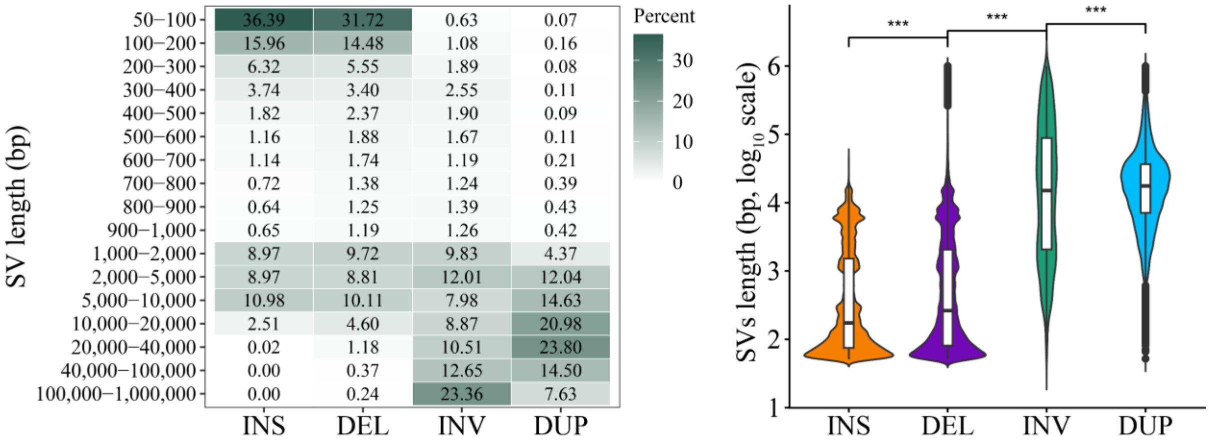

Figure S5. Size distribution of different types of SVs in 21 ONT-sequenced samples (with Wilcoxon's test, *, $P < 0.05$; ***, $P < 0.001$). INS, insertion; DEL, deletion; INV, inversion; DUP, duplication.

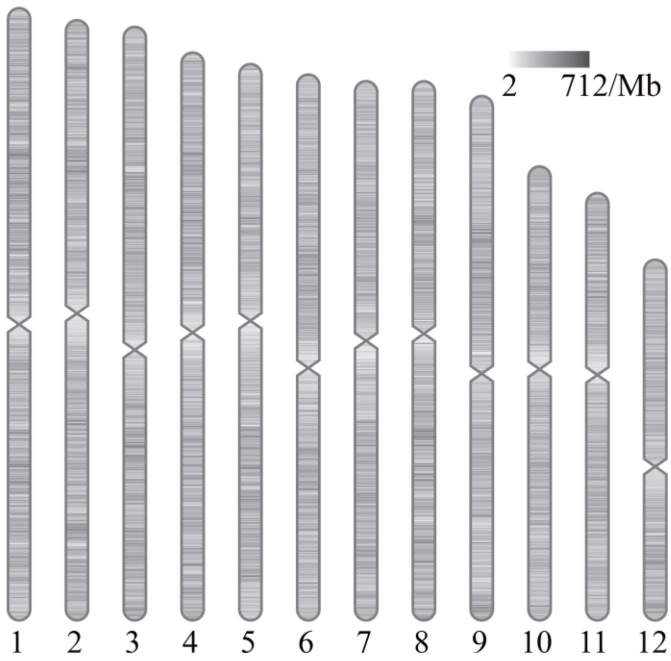

Figure S6. Density of all SVs (~5.7 million) along 12 chromosomes in 1-Mb non-overlapping windows.

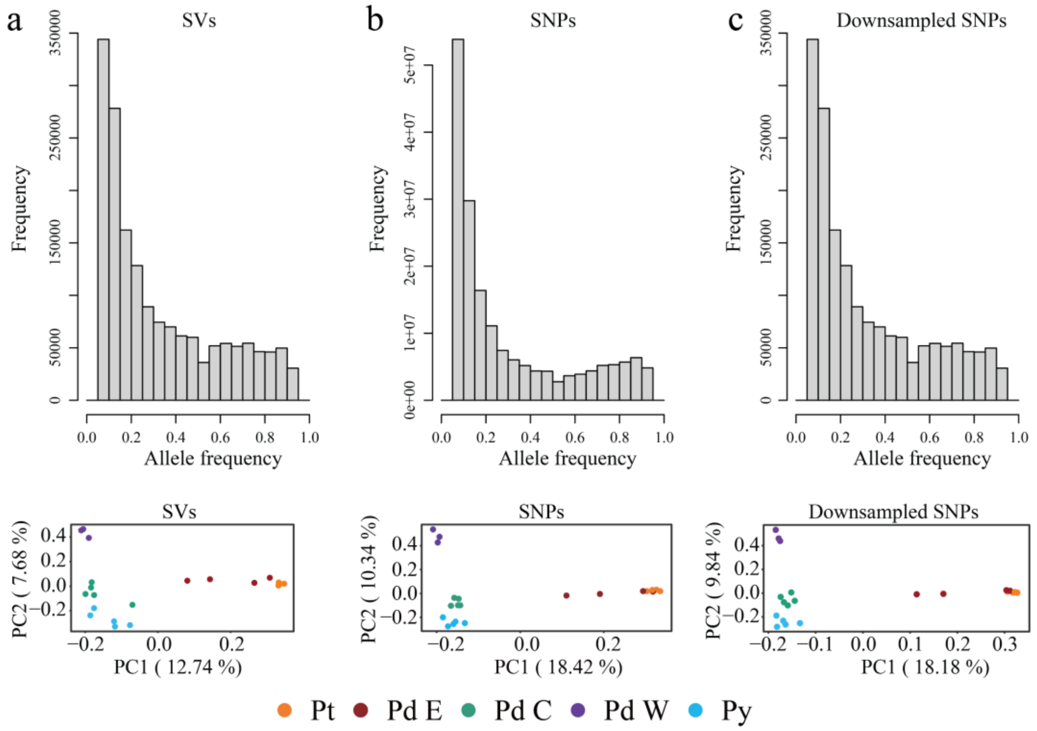

Figure S7. Allele frequency distribution of genome-wide SVs (a), genome-wide SNPs (b), and downsampled SNPs to match the site frequency spectra of SVs (c). Their corresponding PCA plots for the 21 samples are shown in the lower panel.

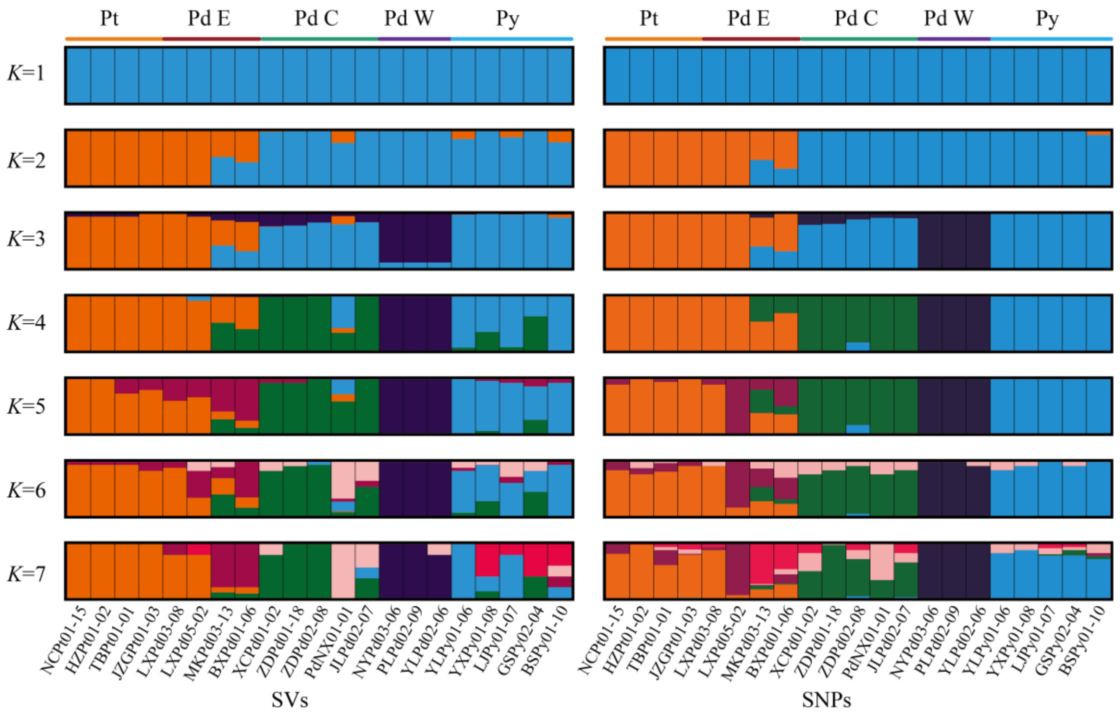

Figure S8. Patterns of ADMIXTURE (*K*=1 to 7) based on SVs and SNPs. Results for $K >$ 7 are not shown due to inconsistent clustering among replicate runs.

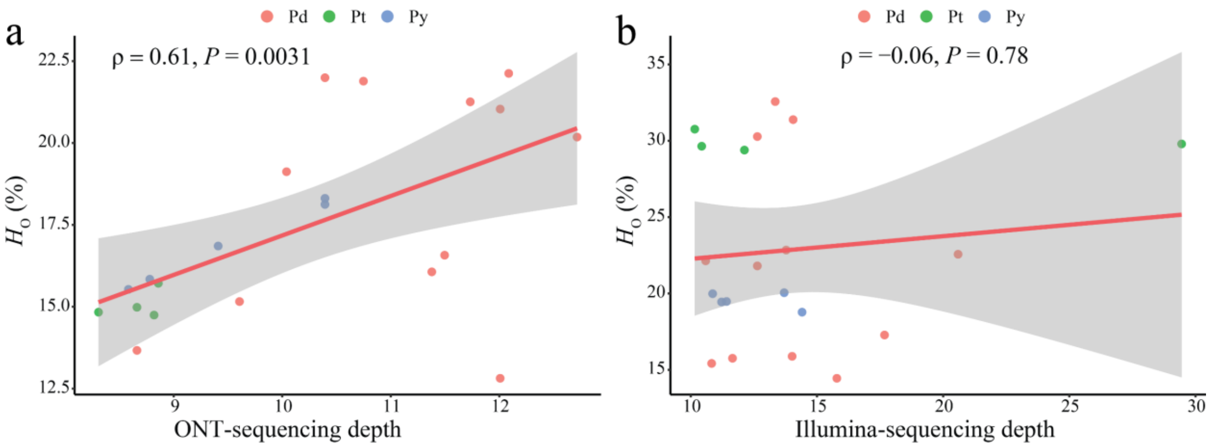

Figure S9. Correlation between sequencing depth and observed individual heterozygosity (*H*O) in SVs and SNPs. (a) ONT sequencing depth versus SV-based *H*O. (b) Illumina sequencing depth versus SNP-based *H*O. Red lines indicate linear regression fits with 95% confidence intervals. Spearman's rank correlation coefficient (ρ) and corresponding *P* values are shown. Pd, *P. densata*; Pt, *Pinus tabuliformis*; Py, *P. yunnanensis*.

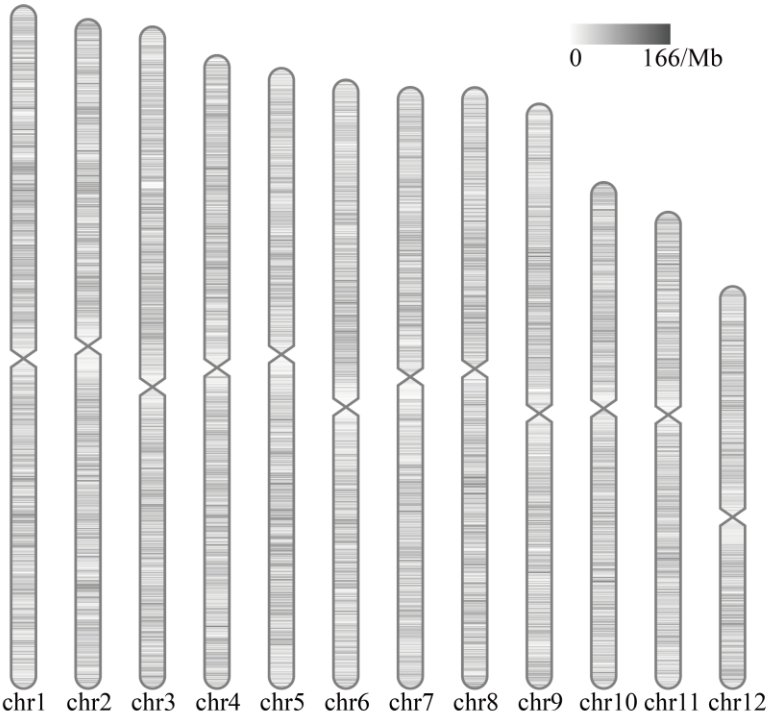

Figure S10. Genome-wide distribution of the SVs (~1 million) shared among all three species, *P. densata, P. tabuliformis* and *P. yunnanensis,* in 1-Mb non-overlapping windows.

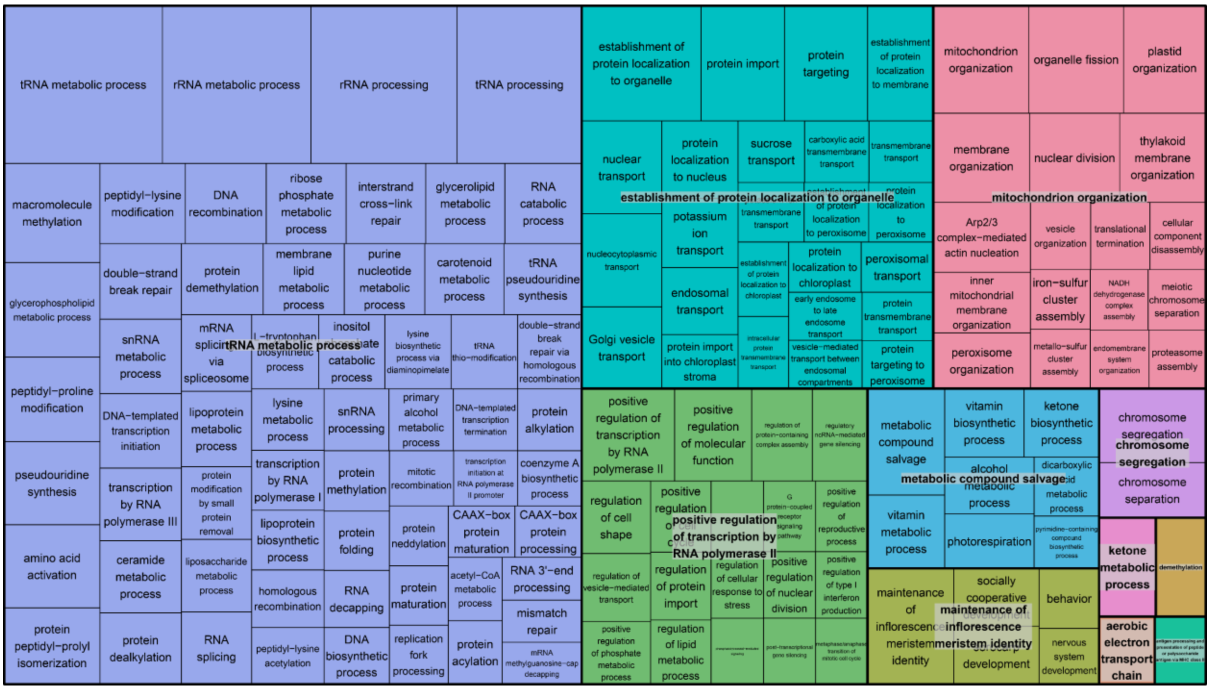

Figure S11. Gene ontology enrichment (biological process) of the genes overlapped with the SVs that were common to all three species, *P. densata, P. tabuliformis* and *P. yunnanensis*.

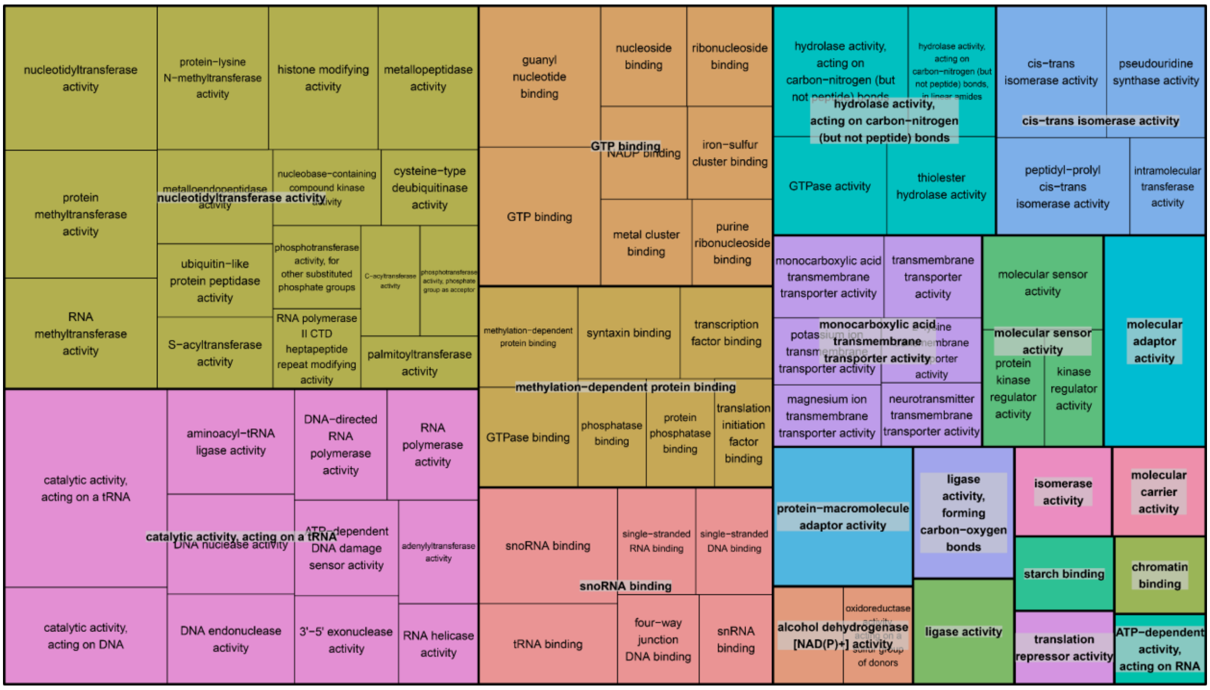

Figure S12. Gene ontology enrichment (molecular function) of the genes overlapped with the SVs that were common to all three species, *P. densata, P. tabuliformis* and *P. yunnanensis*.

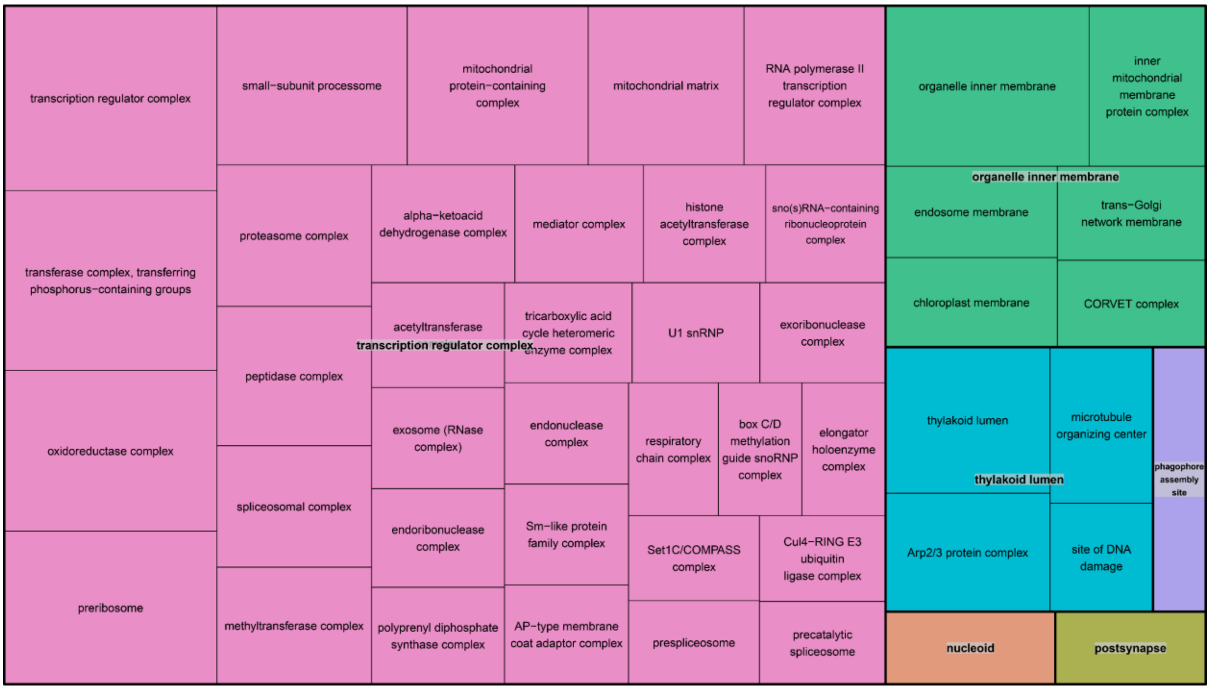

Figure S13. Gene ontology enrichment (cellular component) of the genes overlapped with the SVs that were common to all three species, *P. densata, P. tabuliformis* and *P. yunnanensis*.

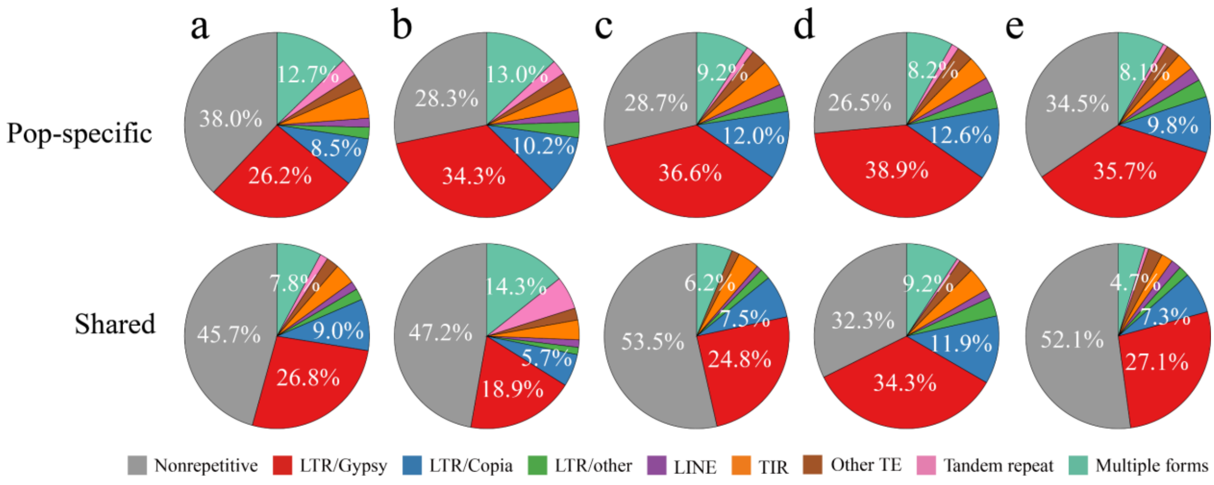

Figure S14. Proportion of population-specific and population-shared SVs associated with different repeat types across five SV categories: (a) insertions (INS), (b) deletions (DEL), (c) inversions (INV), (d) duplications (DUP), and (e) translocations (TRA). Pie charts are color-coded by the types of repeat elements. LTR, long terminal repeat; LINE, long interspersed nuclear element; TIR, terminal inverted repeat; TE, transposable element; Multiple forms, repeats with mixed types.

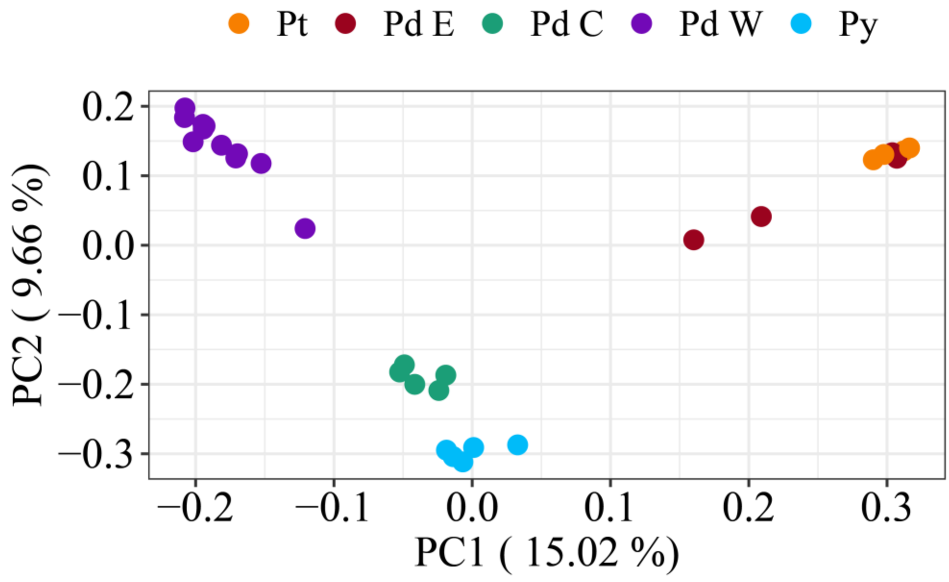

Figure S15. PCA of 29 samples included in this study based on genome-wide SNP data. The sample size of population Pd W was increased to 11 individuals to enable graph-based analyses at the population level.

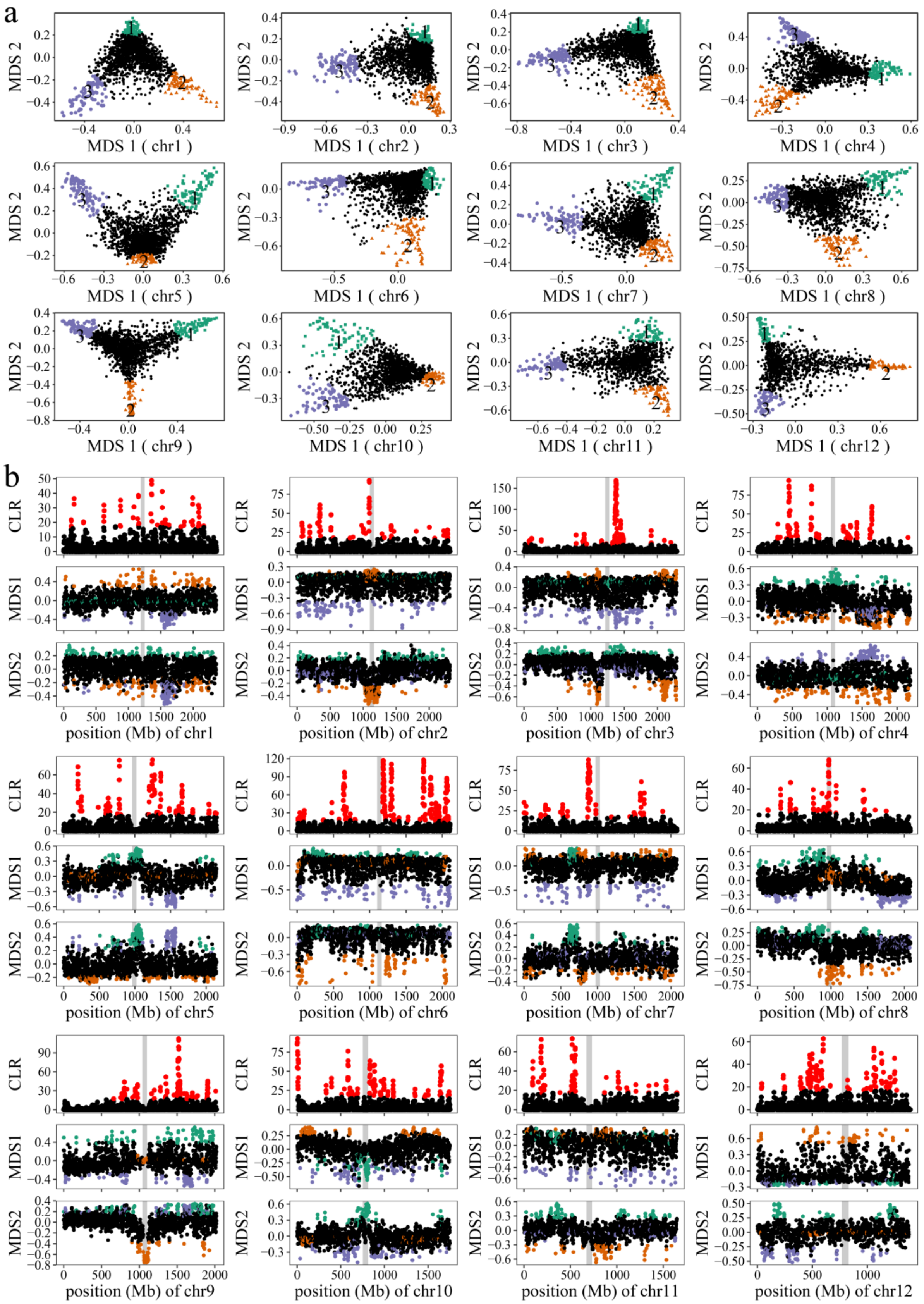

Figure S16. Dot plots of composite likelihood ratio (CLR) scores and multidimensional scaling (MDS) coordinates in the SV dataset of Pd W across all 12 chromosomes. (a) Distribution of genomic windows in two-dimensional MDS space for each chromosome. Green,

orange, and purple dots represent three sets of extreme windows (corners 1, 2, and 3, respectively), each set comprising 5% of all windows. (b) Genome-wide distributions of CLR scores and MDS coordinates. Red dots in CLR plots indicate windows in the genome-wide top 5% of CLR scores. Colors in MDS plots correspond to the three corner outliers shown in (a). Windows identified as outliers by both methods were considered candidate regions under selection. Both analyses were conducted in non-overlapping 1-Mb windows. Grey bars indicate the putative centromeric regions.

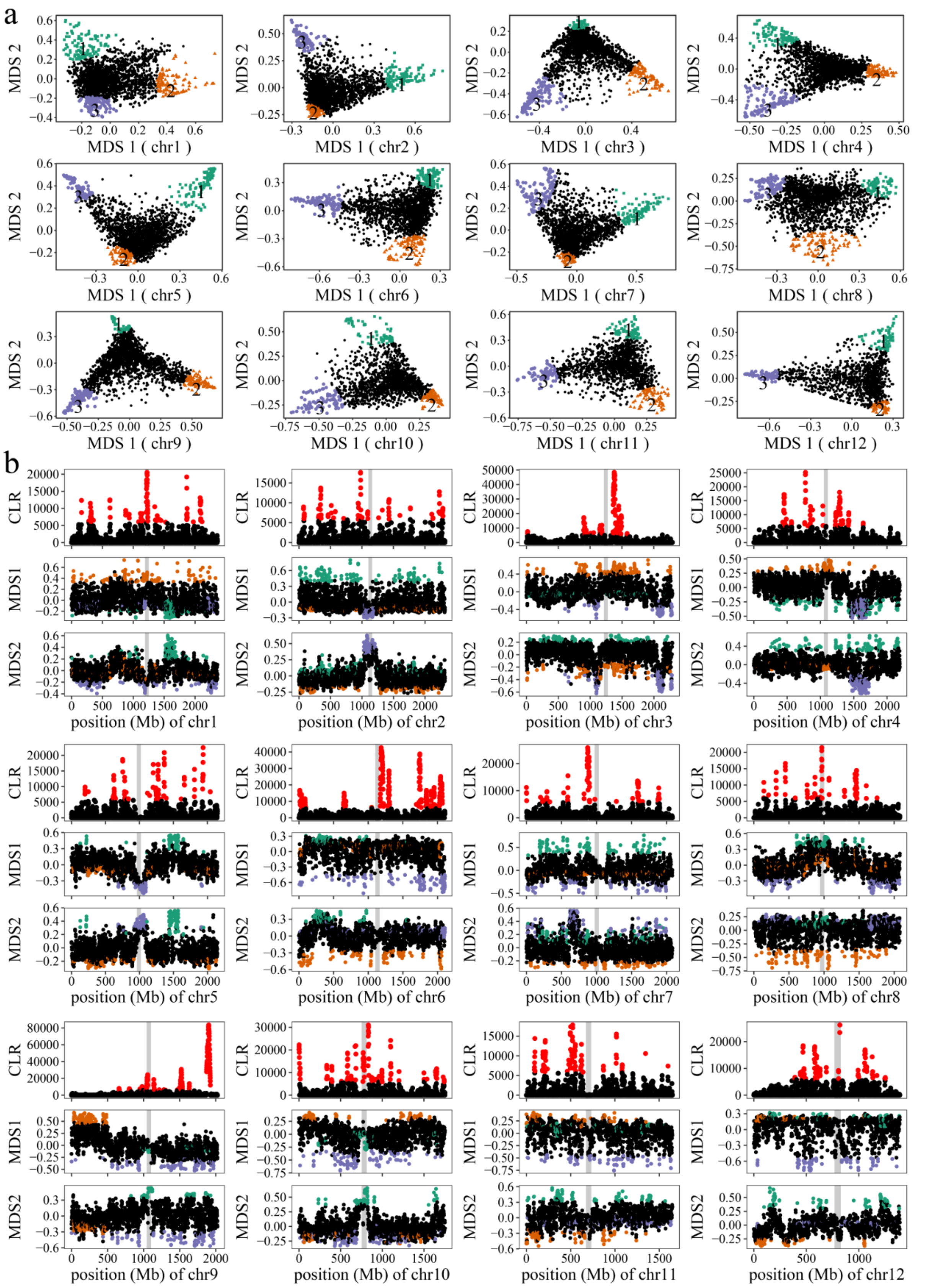

Figure S17. Dot plot of composite likelihood ratio (CLR) scores and multidimensional scaling (MDS) coordinates in the SNP dataset of Pd W across all 12 chromosomes. (a) Distribution of genomic windows in two-dimensional MDS space for each chromosome. Green,

orange, and purple dots represent three sets of extreme windows (corners 1, 2, and 3, respectively), each set comprising 5% of all windows. (b) Genome-wide distributions of CLR scores and MDS coordinates. Red dots in CLR plots indicate windows in the genome-wide top 5% of CLR scores. Colors in MDS plots correspond to the three corner outliers shown in (a). Windows identified as outliers by both methods were considered candidate regions under selection. Both analyses were conducted in non-overlapping 1-Mb windows. Grey bars indicate the putative centromeric regions.

References

Chen J, Liu Y, Liu M, Guo W, Wang Y, He Q, Chen W, Liao Y, Zhang W, Gao Y, et al. Pangenome analysis reveals genomic variations associated with domestication traits in broomcorn millet. *Nat Genet.* 2023:55:2243–2254. doi: 10.1038/s41588-023-01571-z.

Chen S, Wang P, Kong W, Chai K, Zhang S, Yu J, Wang Y, Jiang M, Lei W, Chen X, et al. Gene mining and genomics-assisted breeding empowered by the pangenome of tea plant *Camellia sinensis*. *Nat Plants.* 2023:9:1986–1999. doi: 10.1038/s41477-023-01565-z.

English AC, Menon VK, Gibbs RA, Metcalf GA, Sedlazeck FJ. Truvari: Refined structural variant comparison preserves allelic diversity. *Genome Biol.* 2022:23:271. doi: 10.1186/s13059-022-02840-6.

Hämälä T, Wafula EK, Guiltinan MJ, Ralph PE, dePamphilis CW, Tiffin P. Genomic structural variants constrain and facilitate adaptation in natural populations of *Theobroma cacao*, the chocolate tree. *Proc Natl Acad Sci USA.* 2021:118:e2102914118. doi: 10.1073/pnas.2102914118.

He Q, Tang S, Zhi H, Chen J, Zhang J, Liang H, Alam O, Li H, Zhang H, Xing L, et al. A graph-based genome and pan-genome variation of the model plant *Setaria*. *Nat Genet.* 2023:55:1232–1242. doi: 10.1038/s41588-023-01423-w.

Huang X, Lin X, Zhou P, Tan W, Gao F, Ni Z, Shi T, Bai Y, Ma C, Ma Y, et al. Pangenome analysis reveals structural variations associated with citric acid accumulation in *Prunus mume*. *Plant Biotechnol J.* 2026:24:2623–2641. doi: 10.1111/pbi.70518.

Jayakodi M, Padmarasu S, Haberer G, Bonthala VS, Gundlach H, Monat C, Lux T, Kamal N, Lang D, Himmelbach A, et al. The barley pan-genome reveals the hidden legacy of mutation breeding. *Nature.* 2020:588:284–289. doi: 10.1038/s41586-020-2947-8.

Jiao C, Xie X, Hao C, Chen L, Xie Y, Garg V, Zhao L, Wang Z, Zhang Y, Li T, et al. Pan-genome bridges wheat structural variations with habitat and breeding. *Nature.* 2025:637:384–393. doi: 10.1038/s41586-024-08277-0.

Kang M, Wu H, Liu H, Liu W, Zhu M, Han Y, Liu W, Chen C, Song Y, Tan L, et al. The pan-genome and local adaptation of *Arabidopsis thaliana*. *Nat Commun.* 2023:14:6259. doi: 10.1038/s41467-023-42029-4.

Li W, Chu C, Zhang T, Sun H, Wang S, Liu Z, Wang Z, Li H, Li Y, Zhang X, et al. Pan-genome analysis reveals the evolution and diversity of *Malus*. *Nat Genet.* 2025:57:1274–1286. doi: 10.1038/s41588-025-02166-6.

Liang YY, Liu H, Lin QQ, Shi Y, Zhou BF, Wang JS, Chen XY, Shen Z, Qiao LJ, Niu JW, et al. Pan-genome analysis reveals local adaptation to climate driven by introgression in oak species. *Mol Biol Evol.* 2025:42:msaf088. doi: 10.1093/molbev/msaf088.

Liu Y, Du H, Li P, Shen Y, Peng H, Liu S, Zhou G-A, Zhang H, Liu Z, Shi M, et al. Pan-genome of wild and cultivated soybeans. *Cell.* 2020:182:162–176.e13. doi: 10.1016/j.cell.2020.05.023.

Liu Z, Wang N, Su Y, Long Q, Peng Y, Shangguan L, Zhang F, Cao S, Wang X, Ge M, et al. Grapevine pangenome facilitates trait genetics and genomic breeding. *Nat Genet.* 2024:56:2804–2814. doi: 10.1038/s41588-024-01967-5.

Ma W, Liu Y, Wei X, Zhang X, Li X, Liu Z, Yuan L, Li G, Zhang S, Yang Q, et al. Gapless pangenome analyses reveal fast *Brassica rapa* subspeciation. *Science.* 2026:391:eady7590. doi: 10.1126/science.ady7590.

Meng Q, Xie P, Xu Z, Tang J, Hui L, Gu J, Gu X, Jiang S, Rong Y, Zhang J, et al. Pangenome analysis reveals yield- and fiber-related diversity and interspecific gene flow in *Gossypium barbadense* L. *Nat Commun.* 2025:16:4995. doi: 10.1038/s41467-025-60254-x.

Oren E, Dafna A, Tzuri G, Halperin I, Isaacson T, Elkabetz M, Meir A, Saar U, Ohali S, La T, et al. Pan-genome and multi-parental framework for high-resolution trait dissection in melon (*Cucumis melo*). *Plant J.* 2022:112:1525–1542. doi: 10.1111/tpj.16021.

Qin P, Lu H, Du H, Wang H, Chen W, Chen Z, He Q, Ou S, Zhang H, Li X, et al. Pan-genome analysis of 33 genetically diverse rice accessions reveals hidden genomic variations. *Cell.* 2021:184:3542–3558.e3516. doi: 10.1016/j.cell.2021.04.046.

Shang L, Li X, He H, Yuan Q, Song Y, Wei Z, Lin H, Hu M, Zhao F, Zhang C, et al. A super pan-genomic landscape of rice. *Cell Res.* 2022:32:878–896. doi: 10.1038/s41422-022-00685-z.

Shi T, Zhang X, Hou Y, Jia C, Dan X, Zhang Y, Jiang Y, Lai Q, Feng J, Feng J, et al. The super-pangenome of *Populus* unveils genomic facets for its adaptation and diversification in widespread forest trees. *Mol Plant.* 2024:17:725–746. doi: 10.1016/j.molp.2024.03.009.

Sun H, Zhang J, Liao S, Guo S, Zhou Z, Zhao X, Wu S, Zhao J, Gong G, Wang J, et al. Population-level super-pangenome reveals genome evolution and empowers precision breeding in watermelon. *Nat Genet.* 2026:58:1176–1185. doi: 10.1038/s41588-026-02598-8.

Tang D, Jia Y, Zhang J, Li H, Cheng L, Wang P, Bao Z, Liu Z, Feng S, Zhu X, et al. Genome evolution and diversity of wild and cultivated potatoes. *Nature.* 2022:606:535–541. doi: 10.1038/s41586-022-04822-x.

Yang S, Wang Y, Huang Q, Wang M, Wang S, Fu X, Zhu C, Cheng J, Liu S, Yang Z, et al. A pangenome of maize provides genetic insights into drought resistance. *Nat Genet.* 2025:57:2831–2841. doi: 10.1038/s41588-025-02378-w.

Yildiz G, Zanini SF, Weber S, Kopalli V, Kox T, Abbadi A, Snowdon RJ, Golicz AA. Graphical pangenomics-enabled characterization of structural variant impact on gene expression in *Brassica napus*. *Theor Appl Genet.* 2025:138:91. doi: 10.1007/s00122-025-04867-2.

You Q, Peng Z, Li Z, Jiang Y, Wan P, Zhao Y, Zhao W, Zhang S, Cheng H, Chen C, et al. Sub-pangenome analysis reveals structural variants associated with fruit color and bacterial wilt resistance in eggplant. *Nat Commun.* 2026:17:3075. doi: 10.1038/s41467-026-69764-8.

Zhang X, Lan L, Yang Y, Guan H, Peng D, Jiang H, Wu Q, Huang R, Liao X, Lin S, et al. Pangenomic analyses of rose uncover widespread structure variation and empower genomics-directed breeding. *Nat Genet.* 2026:58:1164–1175. doi: 10.1038/s41588-026-02569-z.

Zhou Y, Zhang Z, Bao Z, Li H, Lyu Y, Zan Y, Wu Y, Cheng L, Fang Y, Wu K, et al. Graph pangenome captures missing heritability and empowers tomato breeding. *Nature.* 2022:606:527–534. doi: 10.1038/s41586-022-04808-9.
